# Antimicrobial bianthrones from the crinoid *Heterometra* sp.†

**DOI:** 10.1039/d4ra05594b

**Published:** 2024-12-02

**Authors:** Vítor F. Freire, Lucero Martínez-Fructuoso, Rohitesh Kumar, Rhone K. Akee, Christopher C. Thornburg, Susan Ensel, Ekene Okoroafor, Jason R. Evans, Dongdong Wang, Brian D. Peyser, Tanja Grkovic, Barry R. O'Keefe

**Affiliations:** a Natural Products Branch, Developmental Therapeutic Program, Division of Cancer Treatment and Diagnosis, National Cancer Institute Frederick Maryland 21702-1201 USA; b Natural Products Support Group, Leidos Biomedical Research, Inc., Frederick National Laboratory for Cancer Research Frederick Maryland 21702-1201 USA; c Department of Chemistry and Physics, Hood College Frederick Maryland 21701-8599 USA; d Molecular Targets Program, Center for Cancer Research, National Cancer Institute Frederick Maryland 21702-1201 USA

## Abstract

Antimicrobial resistance is a global public health problem and identification of new chemical scaffolds is important to overcoming this threat. In a recent high-throughput discovery campaign, fractions derived from the organic extract of crinoid, *Heterometra* sp. (Echinodermata), showed antibacterial activity. Chemical investigation of this extract led to the isolation of three natural products, namely crinemodin bianthrone (1), the new structure 1′′-dehydrocrinemodin bianthrone (2), and 1′′-hydroxycrinemodin bianthrone (3). Their planar structures were determined through HRESIMS and 1D and 2D NMR analysis while a combination of chemical and chirooptical methods was employed to define their absolute configurations for the first time. Variations in proton chemical shifts as well as instability in DMSO and pyridine were observed for 3. Compounds 1 and 3 showed selective antimicrobial activity against Gram-positive bacterial strains.

## Abstract 2

Antimicrobial bianthrones from *Heterometra* sp. and their chemical behaviour.

## Graphical abstract

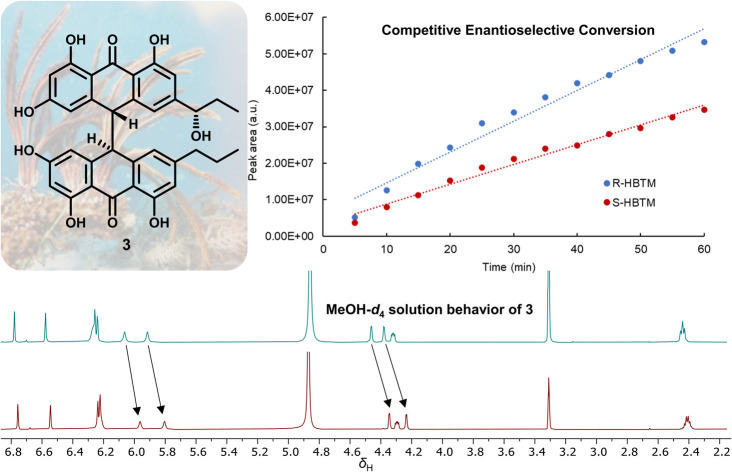

## 1. Introduction

Antimicrobial resistance (AMR) ranks among the top ten global public health issues.^1^ It is estimated that in 2019, bacteria resistant to antibiotics were associated with 4.95 million deaths, and 1.27 million were directly attributable to this cause worldwide.^2^ The Centers for Disease Control and Prevention (CDC) in the United States lists 18 antimicrobial-resistant bacteria and fungi, with vancomycin-resistant *Enterococci* (VRE), multidrug-resistant *Pseudomonas aeruginosa*, drug-resistant *Candida*, and methicillin-resistant *Staphylococcus aureus* (MRSA) classified as serious threats.^3^ Despite the significant burden of AMR, the development of new molecules for its treatment demonstrates a lack of innovation. From 2017 to 2021 a total of 12 new antibacterial drugs were approved. However, among them, only two—Vabomere® and Lefamulin®—introduced new chemical classes.^4^

To encourage screening of diverse chemical libraries against microbial targets, the National Institute of Allergy and Infectious Diseases (NIAID) and the National Cancer Institute (NCI) partnered to perform a high throughput screen of more than 326 000 natural product fractions against three pathogens, including *S. aureus*, *Escherichia coli* (2 strains, including wild type and *tol*C efflux mutant) and *C. albicans*. A proof-of-concept study selected 75 active fractions from this discovery campaign for the isolation and identification of the bioactive metabolites. Briefly, HPLC-based subfractions were generated from 1 mg of material, and active subfractions had their chemotype annotated based on NMR, LC-MS and IR data. As previously reported, two selected extracts from this screen led to the isolation of 2-amino imidazole and pyridoacridine alkaloids with potent and selective antifungal and antibacterial activities.^5^

As an extension to this proof-of-concept study, herein we describe the scale-up isolation and antibiotic activity of the compounds isolated from *Heterometra* sp. (Echinodermata). Partially purified subfractions from the organic extract of *Heterometra* sp. were identified as having selective activity against a Gram-positive bacterium *Staphylococcus aureus* (Fig. S1†).^5^ To fully characterize its active constituent(s), the organic extract (NSC number #C4711) was subjected to multiple steps of chromatographic separation, leading to the isolation of three crinemodin bianthrones 1–3.

## 2. Experimental section

### 2.1. General procedures

Optical rotations were measured on a Rudolph research analytical AUTOPOL IV automatic polarimeter with a 0.25 dm pathlength cell in MeOH at 25 °C. UV spectra were recorded as methanol solutions on a Varian Cary 50-Bio UV/vis spectrophotometer. ECD experiments were recorded as acetonitrile solutions on a J-1500 CD spectropolarimeter using a quartz cell 1 mm path, at room temperature. FTIR spectra were recorded as thin films on a Bruker Alpha II spectrometer. NMR spectra were recorded at 25 °C on either Bruker Avance III HD spectrometer, equipped with a 5 mm TCI Cryo-Probe Prodigy or a Bruker Avance III spectrometer equipped with a 3 mm TCI cryogenic probe, both operating at a frequency of 600 MHz for the ^1^H nucleus and 151 MHz for the ^13^C nucleus. For the 3 mm TCI cryogenic probe, all 2D NMR experiments were acquired with non-uniform sampling (NUS) set to 25% using the standard Bruker pulse sequences. For the 5 mm TCI cryogenic probe, all 2D NMR experiments were acquired with non-uniform sampling (NUS) set to 40% for ^1^H–^1^H detected experiments or 35% for ^1^H–^13^C detected experiments using the standard Bruker pulse sequences. The ^1^H–^13^C HMBC experiments were acquired with ^*n*^*J*_CH_ = 8.0 Hz. Spectra were calibrated to residual solvent signals at *δ*_H_ 3.31 and *δ*_C_ 49.0 (MeOH-*d*_4_). NMR FID processing and data interpretation was done using MestReNova software, version 14.2. High-resolution mass spectra were recorded on an Agilent 1260 Infinity II UHPLC system coupled to an Agilent 6545 QToF equipped with a dual AJS ESI source. Semi-preparative scale HPLC purification was performed with a Gilson HPLC purification system equipped with a GX-281 liquid handler, a 322-binary pump, and a 172-photodiode array detector. All solvents used for chromatography, UV, and MS were HPLC grade, and the H_2_O was Millipore Milli-Q PF filtered.

### 2.2. Collection, extraction, and isolation

The crinoid *Heterometra* sp. was collected in Indo-West Pacific (Northwestern Australia) in August 1988 by the Australian Institute of Marine Science under contract to the Natural Products Branch, Developmental Therapeutics Program, Division of Cancer Treatment and Diagnosis, NCI. The specimen was taxonomically identified by Martin Riddle, and a voucher (Q66C55280) was deposited at the Smithsonian Institution in Suitland, MD. The crinoid (wet weight 316.84 g) was extracted in water, followed by a MeOH/DCM overnight soak according to the Natural Products Branch's standard marine extraction procedure,^6^ to give 5.84 g of the organic extract (C4711). A portion containing 300 mg of C4711 was prefractionated on a C_8_ SPE column (2 g), generating seven fractions:^7^ 95 : 5 H_2_O : MeOH (C4711_1), 80 : 20 H_2_O : MeOH (C4711_2), 60 : 40 H_2_O : MeOH (C4711_3), 40 : 60 H_2_O : MeOH (C4711_4), 20 : 80 H_2_O : MeOH (C4711_5), 0 : 100 H_2_O : MeOH (C4711_6), 50 : 50 MeCN : MeOH (C4711_7).

Fraction C4711_5 (26.5 mg) was submitted for HPLC separation using an Onyx Monolithic C_18_ column (100 × 10 mm, Phenomenex) and mobile phase of H_2_O + 0.1% formic acid (solvent A) and MeCN + 0.1% formic acid (solvent B), with the following gradient conditions at 3.8 mL min^−1^: hold at 70 : 30 (A : B) for 1.5 min, from 70 : 30 to 0 : 100 (A : B) in 7.5 min, hold at 100% (B) for 4.0 min, for a total of 13 min run. Fraction collection was performed in 30 s increments between 1.5 and 12.5 min (22 fractions).^8^ Fractions C4711_5_8 to 17 were combined (11.3 mg) and further separated using a Kinetex C_8_ column (150 × 21.2 mm, 5 μm, 100 Å, Phenomenex) and mobile phase of H_2_O + 0.1% formic acid (solvent A) and MeCN + 0.1% formic acid (solvent B), with the following gradient conditions at 9.0 mL min^−1^: from 50 : 50 to 30 : 70 (A : B) for 32 min, and hold at 30 : 70 (A : B) for 5.0 min. Fraction collection was performed in 30 s increments, yielding crinemodin bianthrone (1.8 mg, 0.6% of organic extract yield; 1) and 1′′-dehydrocrinemodin bianthrone (1.5 mg, 0.5% of organic extract yield; 2).

Fractions C4711_6 and 7 were combined (90.6 mg) and submitted for HPLC separation using an Onyx monolithic C_18_ column (100 × 10 mm, Phenomenex) and mobile phase of H_2_O + 0.1% formic acid (solvent A) and MeCN + 0.1% formic acid (solvent B), with the following gradient conditions at 3.8 mL min^−1^: hold at 70 : 30 (A : B) for 1.5 min, from 70 : 30 to 0 : 100 (A : B) in 7.5 min, hold at 100% (B) for 4.0 min, for a total of 13 min run. Fraction collection was performed in 30 s increments between 1.5 and 12.5 min (22 fractions).^8^ Fractions C4711_6_13 and 14 were combined (5.1 mg) and further separated using a Kinetex C_8_ column (150 × 21.2 mm, 5 μm, 100 Å, Phenomenex) and mobile phase of H_2_O + 0.1% formic acid (solvent A) and MeCN + 0.1% formic acid (solvent B), with the following gradient conditions at 9.0 mL min^−1^: from 50 : 50 to 35 : 65 (A : B) for 32 min, and hold at 35 : 65 (A : B) for 5 min, in a total of 37 min run. Fraction collection was performed in 30 s increments, yielding 1′′-hydroxycrinemodin bianthrone (2.5 mg, 0.83% of organic extract yield; 3).

#### 2.2.1. Crinemodin bianthrone (1)

Brown, amorphous solid; [*α*]_D_^24^ + 85 (*c* 0.09, MeOH); UV (MeOH) *λ*_max_ (log *ε*) 278 (1.72), 362 (1.79) nm; ECD (3.81 × 10^−5^ M, MeCN) *λ*_max_ (Δ*ε*) 265 (−3.1), 300 (+3.4), 345 (−3.6) and 394 (+3.9) nm; IR (film) *ν*_max_ 2961, 2933, 2873, 1619, 1602, 1561, 1486, 1377, 1339, 1294, 1249, 1165, 1062, 918, 854, 790, 755, 682, 550 cm^−1^; ^1^H and ^13^C data (MeOH-*d*_4_) in Table 1; HRESIMS *m*/*z* 567.2015 [M + H]^+^ (calcd for C_34_H_31_O_8_^+^*m*/*z* 567.2014, *Δ* = 0.17 ppm).

**Table 1:** NMR data for bianthrones 1 and 2 in MeOH-*d*_4_

Position	1		2	
	*δ*_C_, type^a^	*δ*_H_, mult^b^ (*J* in Hz)	*δ*_C_, type^a^	*δ*_H_, mult^b^ (*J* in Hz)
1	102.9, CH	6.45, br s	109.8, CH	6.41, s
2	165.5, C		165.6, C	
3	109.7, CH	6.28, d (2.2)	103.0, CH	6.26, s
4	166.5, C		166.5, C	
5	111.9, C		111.8, C	
6	191.6, C		191.4, C	
7	115.1, C		114.7, C	
8	162.8, C		163.1, C	
9	117.0, CH	6.54, s	114.9, CH	6.55, s
10	151.8, C		145.2, C	
11	121.6, CH	5.72, s	118.1, CH	5.78, s
12	140.7, C		140.6, C	
13	57.4, CH	4.37, s	57.4, CH	4.27, d (2.9)
14	146.9, C		146.9, C	
1′	102.9, CH	6.45, br s	109.7, CH	6.41, s
2′	165.5, C		165.5, C	
3′	109.7, CH	6.28, d (2.2)	102.9, CH	6.26, s
4′	166.5, C		166.5, C	
5′	111.9, C		111.8, C	
6′	191.6, C		191.2, C	
7′	115.1, C		115.4, C	
8′	162.8, C		162.8, C	
9′	117.0, CH	6.54, s	116.9, CH	6.50, s
10′	151.8, C		151.7, C	
11′	121.6, CH	5.72, s	121.4, CH	5.65, s
12′	140.7, C		140.6, C	
13′	57.4, CH	4.37, s	57.3, CH	4.17, d (2.9)
14′	146.9, C		146.7, C	
1′′	39.2, CH_2_	2.39, t (7.5)	131.0, CH	6.16, d (15.4)
2′′	24.4, CH_2_	1.54, m	131.6, CH_2_	6.13, m
3′′	14.3, CH_3_	0.94, t (7.3)	18.7, CH_3_	1.87, d (5.9)
1′′′	39.2, CH_2_	2.39, t (7.5)	39.2, CH_2_	2.36, t (7.8)
2′′′	24.4, CH_2_	1.54, m	24.4, CH_2_	1.51, m
3′′′	14.3, CH_3_	0.94, t (7.3)	14.3, CH_3_	0.92, t (7.7)

a 151 MHz.

b 600 MHz.

#### 2.2.2. 1′′-Dehydrocrinemodin bianthrone (2)

Brown, amorphous solid; [*α*]_D_^24^ + 64 (*c* 0.07, MeOH); UV (MeOH) *λ*_max_ (log *ε*) 282 (2.10), 366 (2.19) nm; ECD (3.94 × 10^−5^ M, MeCN) *λ*_max_ (Δ*ε*) 273 (−5.2), 303 (+2.5), 342 (−5.3) and 406 (+4.9) nm; IR (film) *ν*_max_ 2926, 2853, 1617, 1602, 1474, 1376, 1340, 1291, 1251, 1206, 1063, 964, 853, 760 cm^−1^; ^1^H and ^13^C data (MeOH-*d*_4_) in Table 1; HRESIMS *m*/*z* 565.1860 [M + H]^+^ (calcd for C_34_H_29_O_8_^+^*m*/*z* 565.1857, *Δ* = 0.53 ppm).

#### 2.2.3. 1′′-Hydroxycrinemodin bianthrone (3)

Brown, amorphous solid; [*α*]_D_^24^ + 133 (*c* 0.15, MeOH); UV (MeOH) *λ*_max_ (log *ε*) 277 (4.25), 363 (4.27) nm; ECD (2.75 × 10^−5^ M, MeCN) *λ*_max_ (Δ*ε*) 272 (−1.3), 306 (+1.8), 348 (−0.8) and 395 (+0.8) nm; IR (film) *ν*_max_ 2964, 2931, 2874, 1600, 1563, 1483, 1466, 1364, 1337, 1290, 1240, 1163, 1064, 967, 917, 853, 789, 752, 647, 590, 550 cm^−1^; HRESIMS *m*/*z* 605.1785 [M + Na]^+^ (calcd for C_34_H_30_O_9_Na^+^*m*/*z* 605.1783, *Δ* = 0.33 ppm).

### 2.3. Competitive enantioselective conversion (CEC)

The CEC reaction followed by LC-MS analysis was based on previously described procedure by Lee *et al*.^9^ Compound 3 (0.25 mg, 0.43 μmol) was transferred to two different vials, and dimethylformamide (90 μL) was added as organic solvent. The catalysts *S*- and *R*-HBTM (10.0 μL, 12.9 μmol) were added to their respective vials, along with *N*,*N*-diisopropylethylamine (2.2 μL, 12.9 μmol). Propionic anhydride (1.7 μL, 12.9 μmol) was added to initiate the reaction. Aliquots of 2 μL were taken every 5 min and quenched with 100 μL of MeOH prior to LC-MS analysis, for a total reaction time of 60 min.

Aliquots (5 μL) of the samples collected at different time intervals were injected on an Agilent 1260 Infinity II HPLC system coupled to an Agilent 6230 ToF equipped with a dual AJS ESI source. A Kinetex C_18_ column (50.0 × 2.1 mm, 5 μm, Phenomenex) was used. The mobile phase consisted of H_2_O + 0.1% formic acid (A) and MeCN + 0.1% formic acid (B). The gradient used was maintained at 95 : 5 (A : B) for 1.0 min, from 95 : 5 to 0 : 100 (A : B) for 8.0 min, maintained at 0 : 100 (A : B) for 1.0 min, from 0 : 100 to 95 : 5 (A : B) for 0.5 min and then equilibrated in a post-run at 95 : 5 (A : B) during 1.5 min. The positive mode ESI conditions were 3.5 V of capillary voltage, 1.5 kV of nozzle voltage, gas temperature at 325 °C and gas flow of 10 L min^−1^. The MS spectra were acquired using positive mode, mass range of *m*/*z* 100–3000 and scan rate of 2 spectra per s. The reaction rates were determined by measuring the peak areas of the fully acylated derivative of compound 3, using the extracted ion chromatogram (EIC) for the sodiated molecule [M + Na]^+^ at *m*/*z* 997.3617 (±20 ppm) on MassHunter Qualitative Analysis 10.0 software.

### 2.4. Antimicrobial assay

Microbes, growth, and testing conditions were identical to those used for the primary screen.^5^

### 2.5. Anti-proliferative assay

Purified compounds were tested for cytotoxicity in the recently described HTS384 NCI60 screen.^10^

## 3. Results

### 3.1. Structure elucidation

**Fig. 1:**
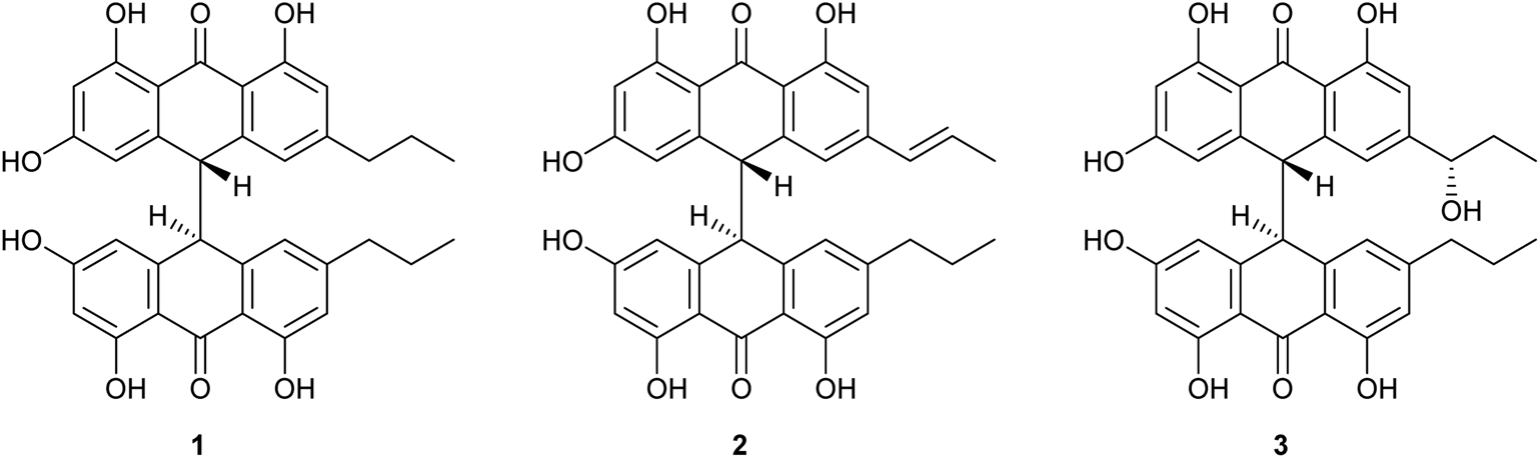
Structures of crinemodin bianthrones (1–3) isolated from *Heterometra* sp.

Crinemodin bianthrone (1) has been previously reported as a constituent of the crinoid *Lamprometra palmata* by Rideout and Sutherland (1985),^11^ where its structure elucidation was achieved by a combination of chemical degradation, chemical synthesis, and comparison of *R*_f_ on TLC, and ^1^H NMR data. Here, we present full spectrometric and spectroscopic characterization of 1, achieved using HRESIMS, 1D and 2D NMR, and chiroptical methods. HRESIMS analysis showed a protonated molecule [M + H]^+^ at *m*/*z* 567.2015, corresponding to the molecular formula C_34_H_31_O_8_^+^. The UV spectrum of 1 showed *λ*_max_ of 278 and 362 nm, consistent with the UV spectra of bianthrones.^12,13^ Inspection of the ^1^H NMR spectrum of 1 demonstrated eight proton resonances, while ^13^C NMR spectrum showed seventeen carbon resonances, indicating symmetry in the molecule (Table 1). The bianthrone core was assigned using ^1^H–^13^C HSQC, ^1^H–^13^C HMBC correlations and comparison with literature values.^14^ The propyl group was connected to the bianthrone core through ^1^H–^13^C HMBC correlations from H-1′′/1′′′ (*δ*_H_ 2.39) to C-9/9′ (*δ*_C_ 117.0), C-10/10′ (*δ*_C_ 151.8) and C-11/11′ (*δ*_C_ 121.6), completing the planar structure of 1. Due to an inner plane of symmetry in 1, it is possible to categorize the stereoisomers of this homobianthrone by their relative configuration as either meso (*cis*) or racemic (*trans*).^11,14–16^ The chiroptical properties of 1 allowed us to establish its relative configuration as *trans*, in agreement with that assigned by Rideout and Sutherland.^11^ Comparison of the experimental ECD spectrum of 1 (Fig. S36†) to reported *in silico* spectra of two *trans*-bianthrone series, allianthrones B and C^15^ and brevianthrones A1 and B1,^12^ based on the Cotton effects at *λ*_max_ 265 nm (Δ*ε* −3.1), 300 (Δ*ε* +3.4) and 345 (Δ*ε* −3.6), indicated the absolute configuration of 1 to be 13*S*,13′*S* (Fig. 1).

**Fig. 2:**
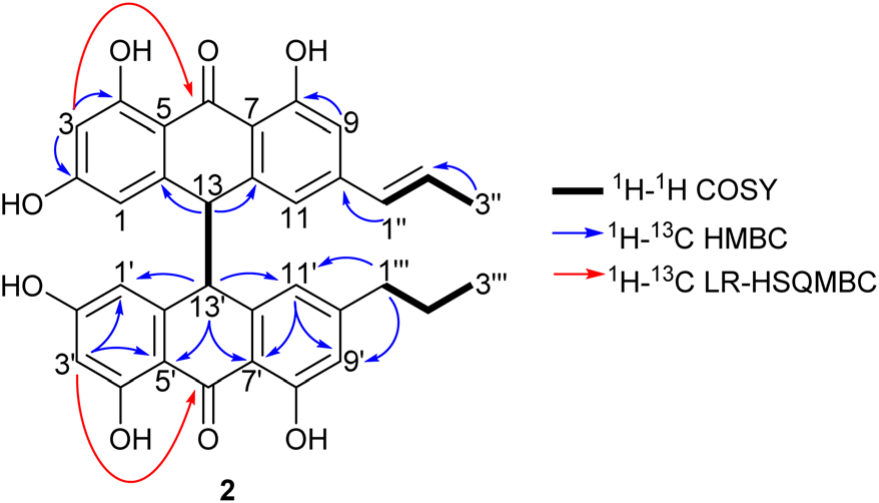
Key 2D NMR correlations used to establish the structure of 1′′-dehydrocrinemodin bianthrone (2).

1′′-Dehydrocrinemodin bianthrone (2) showed a protonated molecule [M + H]^+^ at *m*/*z* 565.1860 by HRESIMS, corresponding to the molecular formula C_34_H_29_O_8_^+^. This mass difference and the absence of symmetry observed in the ^1^H and ^13^C NMR data, suggested 2 contained an unsaturated alkyl group relative to 1. Analysis of 1D and 2D NMR data revealed that the bianthrone core of the molecule was identical to that of 1. The bianthrone motif was fully characterized with the aid of the ^1^H–^13^C LR-HSQMBC^17^ experiment that showed a ^4^*J*_CH_ correlation from H-3/3′ (*δ*_H_ 6.26) to the C-6/6′ (*δ*_C_ 191.4/191.2) carbonyl. Inspection of additional 2D NMR data revealed one side chain composed of a saturated propyl group, H-1′′′ (*δ*_H_ 2.36), H-2′′′ (*δ*_H_ 1.51) and H-3′′′ (*δ*_H_ 0.92), connected to the bianthrone core at C-10′ (*δ*_C_ 151.7) (Fig. 2), identical to that of 1. The ^1^H–^1^H COSY spectrum of the other side chain revealed a contiguous spin system between H-1′′ (*δ*_H_ 6.16), H-2′′ (*δ*_H_ 6.13) and H-3′′ (*δ*_H_ 1.87), indicative of a propenyl group. The ^1^H–^13^C HMBC correlations between H-1′′ and H-2′′ to C-10 (*δ*_C_ 145.2) indicated the connection of the propenyl group to the bianthrone core *via* C-10. The ^1^H NMR spectrum showed that H-1′′ (*δ*_H_ 6.16) had a coupling constant of 15.4 Hz, indicating a *trans* geometry of the double bond. Upon comparison of the specific rotation and ECD spectra of 2 with that of 1 (Fig. S36†), the absolute configuration of 2 was established to be 13*S*,13′*S*. The structure of 2 was therefore assigned to be a new natural product, named 1′′-dehydrocrinemodin bianthrone.

**Fig. 3:**
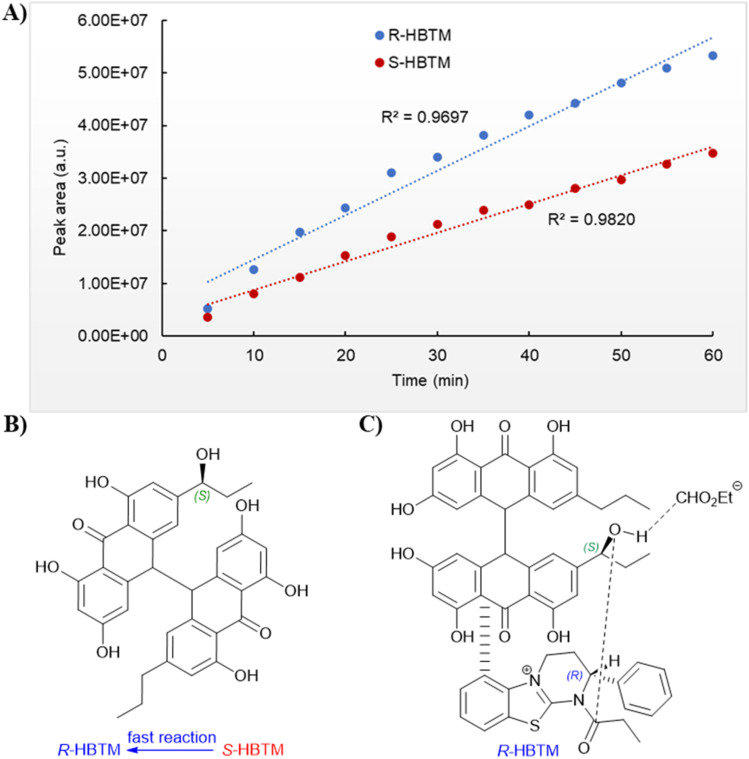
Competitive enantioselective conversion (CEC) reaction with *R*- and *S*-homobenzotetramisole (HBTM) coupled to LC-MS. (A) Reaction rates of 3 treated with propionic anhydride using *R*- and *S*-HBTM catalysts. (B) Adapted mnemonic from Lee *et al.*^9^ used to predict the configuration of secondary alcohols in CEC reaction. (C) Proposed transition state of 3 in CEC reaction.

An additional natural product, 1′′-hydroxycrinemodin bianthrone (3), previously reported from the crinoids *Lamprometra palmata*^11^ and *Himerometra magnipinna*,^14^ was isolated from the same active fraction. While the planar structure of 3 agreed with that previously described,^14^ the absolute configuration was never reported for either the bianthrone core or the secondary alcohol on the side chain. Thus, we used the specific rotation and Cotton effects in the ECD spectrum to assign the bianthrone core of 3 as 13*S*,13′*S*, identical to that of 1 and 2 (Fig. S36†). Due to the insignificant influence of the secondary alcohol at CH-1′′ in the ECD spectrum, chemical methods were used to establish its configuration. The well-established Mosher's method was not successful due to instability of 3 in pyridine-*d*_5_. Alternatively, we applied a competitive enantioselective conversion (CEC) reaction using *R*- and *S*-homobenzotetramisole (HBTM) catalysts followed by LC-MS analysis to establish the absolute configuration of the secondary alcohol.^9,18,19^ This method relies on the kinetic resolution of the acylation of a secondary alcohol by the enantiomeric pair of *R*- and *S*-HBTM catalysts. Two parallel reactions were performed, treating compound 3 with the *R*- or *S*- catalyst. Aliquots of the reactions were taken every 5 min and quenched with MeOH over a period of 60 min. LC-MS data was acquired and the area under the chromatographic peak was integrated using the extracted ion chromatogram (EIC) of the sodium adduct [M + Na]^+^ for the fully acylated product of 3. Analytical curves were plotted and the reaction using *R*-HBTM was established as the fastest one (Fig. 3). The mnemonic used for the determination of the absolute configuration of the secondary alcohol^9,19^ states that compound 3 should be drawn with the polar (π) group on the left side and the alkyl group on the right side. Since *R*-HBTM was the faster reaction, the hydroxy group is positioned up and forward allowing us to establish the absolute configuration of C-1′′ to be *S* for compound 3. Therefore, the absolute configuration of 3 was determined to be 13*S*,13′*S*,1′′*S*.

### 3.2. Solution behavior of bianthrones

**Fig. 4:**
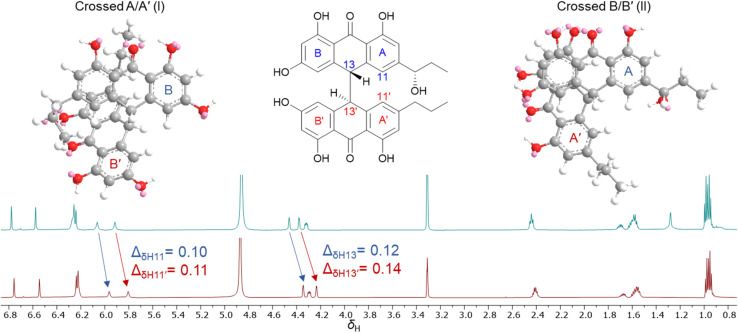
^1^H NMR spectra comparison of 3 acquired in MeOH-*d*_4_ on different days showing the variation of proton chemical shifts.

During isolation and NMR data acquisition, instability of bianthrones 1–3 was observed when exposed to DMSO, DMSO-*d*_6_ and pyridine-*d*_5_. Previous reports indicated that the bianthrones can suffer polymerization^15^ or cleavage to its monomers,^20^ which can be triggered by changes in pH or pressure, and exposure to UV or organic solvents.^15,20^ However, while unstable in some organic solvents, compounds 1–3 showed stability in methanol, water, and acetonitrile which allowed the full assignment of their structure, and revealed unexpected solution behavior as outlined below. Two different fractions containing compound 3 with consistent retention time, MS^1^ spectra, UV profile, [α]_D_ value, and ECD spectra, showed significant differences in *δ*_H_ chemical shifts, especially for two methine doublets at *δ*_H_ 4.2–4.6 (H-13/13′) and the two singlets at *δ*_H_ 6.0–5.8 (H-11/11′). Subsequently, the ^1^H NMR spectrum of the same sample acquired several days apart showed variation of the proton chemical shifts (Fig. 4). The relative level of water in the MeOH-*d*_4_ solution in the NMR tube was speculated to be responsible for the observed behavior. To test this hypothesis, we performed a titration by incrementally adding D_2_O to a solution of 3 in MeOH-*d*_4_. First, 3 was dried under high vacuum overnight, and then solubilized using MeOH-*d*_4_ (99.9%, ampule). A ^1^H NMR spectrum was initially acquired with neat MeOH-*d*_4_, followed by titration with D_2_O (1–20 μL) directly into the NMR tube and subsequent acquisition of additional ^1^H NMR spectra to determine any measurable changes. The addition of D_2_O to the sample caused the proton chemical shifts of 3 to change, especially for H-13/13′ and H-11/11′ (Fig. S37†). We hypothesize that a shielding effect at positions H-13/13′ and H-11/11′ is taking place, due to the higher population of crossed A/A′ (I) gauche conformers of 3 when solvated by D_2_O in solution, compared to the higher population of crossed B/B′ (II) conformers when D_2_O is not present. Although further studies may be required to understand the dynamics of these bianthrones in solution, researchers should be cautious on relying exclusively on ^1^H NMR chemical shifts for configurational assignments of this group of natural products.

### 3.3. Biological activity

The crinemodin bianthrones (1–3) were tested against a range of pathogenic bacterial and fungal strains (Table 2), which included *E. coli* wild-type (BW25113) and *E. coli* efflux-(JW5503-1), *P. aeruginosa* (PAM12626), *S. aureus* (ATCC29213), *E. faecalis* wild-type (ATCC29212), vancomycin-resistant *E. faecium* (VRE, ATCC 700221), *C. albicans* (ATCC90028), and *A. fumigatus* (ATCC MYA-3626), as well as a range of human tumor cell lines in the NCI-60 cell lines screen. Compound 1 was the most active, with MIC values of 5.0, 2.5 and 2.5 mg L^−1^ against the Gram-positive strains *S. aureus*, *E. faecalis* and *E. faecium*, respectively. Compound 3 showed MIC values of 10.0 and 20.0 mg L^−1^ against *S. aureus* and *E. faecalis*, while 2 did not show activity against any of the tested strains. These results suggest that the side chains of the bianthrones are important features for activity against Gram-positive bacteria, with the saturated aliphatic chain exhibiting the highest activity. Notably, no antimicrobial activity was observed for compounds 1–3 against any of the Gram-negative bacteria, the fungal strains tested, or any of the NCI-60 cell lines, demonstrating selectivity of this chemotype for Gram-positive bacterial strains.

**Table 2:** Minimum inhibitory concentration (mg L^−1^) of pure compounds (1–3) against selected bacterial and fungal strains

		*E. coli* WT	*E. coli* efflux	*P. aeruginosa* efflux	*S. aureus* WT	*E. faecalis* WT	*E. faecium* VRE	*C. albicans* WT	*A. fumigatus* WT
1	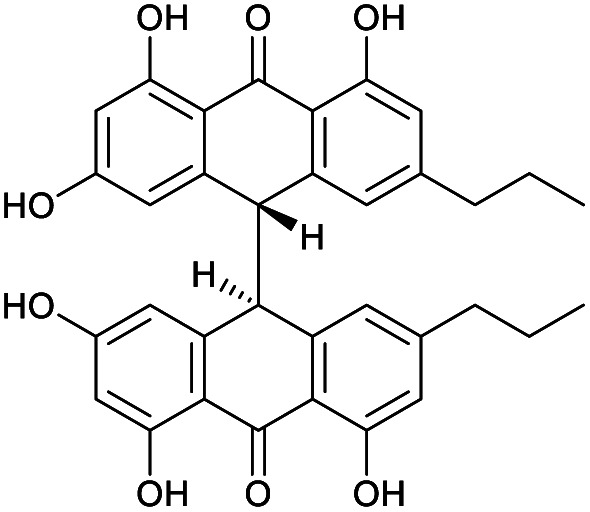	>20	>20	>20	5	2.5	2.5	>10	>10
2	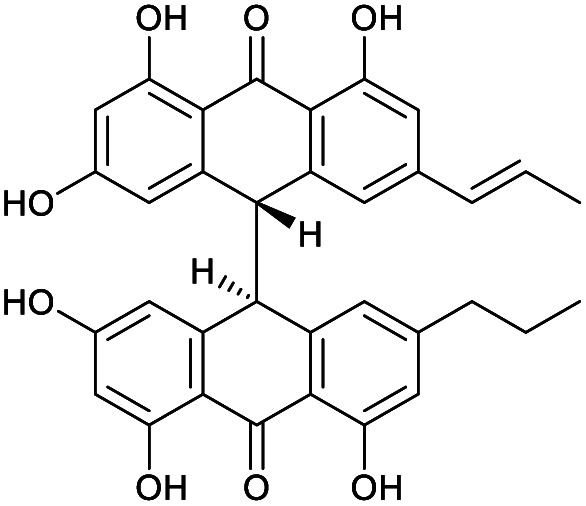	>20	>20	>20	>20	>20	>20	>10	>10
3	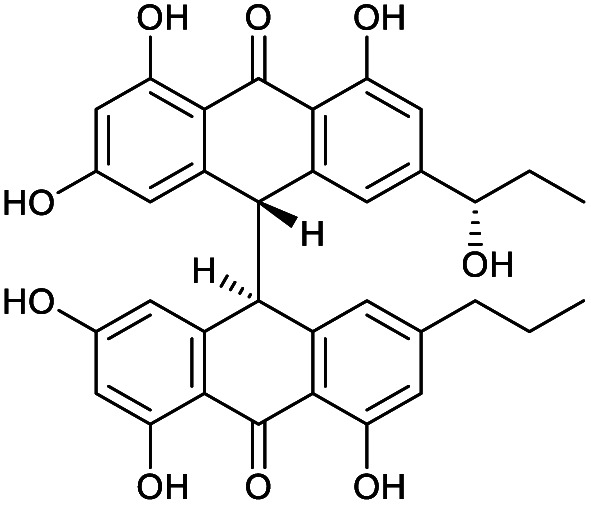	>20	>20	>20	10	20	>20	>10	>10

## 4. Conclusions

Bianthrones 1–3 were isolated from the organic extract of *Heterometra* sp., with 1′′-dehydrocrinemodin bianthrone (2) being a new natural product. For the first time the absolute configuration of crinemodin bianthrones were established by a combination of modern chemical and spectroscopic approaches, enabling the complete configurational assignment of the bianthrone core and side chains. The competitive enantioselective conversion method proved to be a good alternative to solve the absolute configuration of the secondary alcohol in 3. The bianthrones showed interesting solution behavior, which should be taken in consideration when working with this chemotype. The isolated compounds presented selective activity against Gram-positive strains, with 1 showing greater activity against *E. faecalis* wild-type and vancomycin resistant *E. faecium* strains.

## Data availability

The data that support the findings of the present study are available in ESI† of this article. In addition, the raw HRMS and NMR data for the natural products 1–3 has been deposited in the Harvard Dataverse (https://dataverse.harvard.edu/) and can be found at https://doi.org/10.7910/DVN/RJOMB5.

## Conflicts of interest

The authors declare no conflict of interest.

## Supplementary Material

RA-014-D4RA05594B-s001
