# Supplementary material for: Antimicrobial bianthrones from the crinoid Heterometra sp
Source: RSC Adv. 2024 Dec 2;14(51):38200–7. doi: 10.1039/d4ra05594b (PMC11610039; doi:10.1039/d4ra05594b)
Supplement: RA-014-D4RA05594B-s001 [file RA-014-D4RA05594B-s001.pdf]

Supplementary Information (SI) for RSC Advances.
This journal is © The Royal Society of Chemistry 2024

Supplementary Information
for
Antimicrobial bianthrones from the crinoid *Heterometra* sp.

Vítor F. Freire,[a] Lucero Martínez-Fructuoso,[a] Rohitesh Kumar,[b] Rhone K. Akee,[b] Christopher C. Thornburg,[b] Susan Ensel,[b,c] Ekene Okoroafor,[b] Jason R. Evans,[a] Dongdong Wang,[d] Brian D. Peyser,[a] Tanja Grkovic,*[a,d] Barry R. O'Keefe*[a,d]

[a]Natural Products Branch, Developmental Therapeutic Program, Division of Cancer Treatment and Diagnosis, National Cancer Institute, Frederick, Maryland 21702-1201, United States

[b]Natural Products Support Group, Leidos Biomedical Research, Inc., Frederick National Laboratory for Cancer Research, Frederick, Maryland 21702-1201, United States

[c]Department of Chemistry and Physics, Hood College, Frederick, Maryland 21701-8599, United States
[d]Molecular Targets Program, Center for Cancer Research, National Cancer Institute, Frederick, Maryland 21702-1201, United States

LIST OF FIGURES

Figure S1. Screening of subfractions generatedfor the an antimicrobial campaing between NIAID and NCI.[1] 5
Figure S2. HRESIMS spectrum of crinemodin bianthrone (**1**). 6
Figure S3. Expansion of HRESIMS spectrum of crinemodin bianthrone (**1**).......... 6
Figure S4. 600 MHz ^{1}H NMR spectrum of crinemodin bianthrone (**1**) in MeOH-*d*$_4$. The spectrum was acquired over 4 scans using the standard zg30 Bruker pulse sequence. 7
Figure S5. Expansion of 600 MHz ^{1}H NMR spectrum of crinemodin bianthrone (**1**) in MeOH-*d*$_4$. The spectrum was acquired over 4 scans using the standard zg30 Bruker pulse sequence.......... 8
Figure S6. Expansion of 600 MHz ^{1}H NMR spectrum of crinemodin bianthrone (**1**) in MeOH-*d*$_4$. The spectrum was acquired over 4 scans using the standard zg30 Bruker pulse sequence.......... 9
Figure S7. Expansion of 600 MHz ^{1}H NMR spectrum of crinemodin bianthrone (**1**) in MeOH-*d*$_4$. The spectrum was acquired over 4 scans using the standard zg30 Bruker pulse sequence.......... 10

Figure S8. 151 MHz ^{13}C NMR spectrum of crinemodin bianthrone (**1**) in MeOH-*d*4. The spectrum was acquired over 10000 scans using the standard zgpg30 Bruker pulse sequence....... 11

Figure S9. ^{1}H–^{13}C HSQC NMR spectrum of crinemodin bianthrone (**1**) in MeOH-*d*4. The spectrum was acquired over 80 scans at 1024 t1 increments using the standard hsqcedetgpsisp2.3 Bruker pulse sequence....... 12

Figure S10. ^{1}H–^{1}H COSY NMR spectrum of crinemodin bianthrone (**1**) in MeOH-*d*4. The spectrum was acquired over 8 scans at 1024 t1 increments using the standard cosygpppqf Bruker pulse sequence. 13

Figure S11. ^{1}H–^{13}C HMBC NMR spectrum of crinemodin bianthrone (**1**) in MeOH-*d*4. The spectrum was acquired over 296 scans at 2048 t1 increments using the standard hmbcetgpl3nd Bruker pulse sequence. 14

Figure S12. FTIR spectrum of crinemodin bianthrone (**1**)....... 15

Figure S13. HRESIMS spectrum of 1''–dehydrocrinemodin bianthrone (**2**). 16

Figure S14. Expansion of HRESIMS spectrum of 1''–dehydrocrinemodin bianthrone (**2**). 16

Figure S15. 600 MHz ^{1}H NMR spectrum of 1''–dehydrocrinemodin bianthrone (**2**) in MeOH-*d*4. The spectrum was acquired over 4 scans using the standard zg30 Bruker pulse sequence....... 16

Figure S16. Expansion of 600 MHz ^{1}H NMR spectrum of 1''–dehydrocrinemodin bianthrone (**2**) in MeOH-*d*4. The spectrum was acquired over 4 scans using the standard zg30 Bruker pulse sequence....... 17

Figure S17. Expansion of 600 MHz ^{1}H NMR spectrum of 1''–dehydrocrinemodin bianthrone (**2**) in MeOH-*d*4. The spectrum was acquired over 4 scans using the standard zg30 Bruker pulse sequence....... 17

Figure S18. Expansion of 600 MHz ^{1}H NMR spectrum of 1''–dehydrocrinemodin bianthrone (**2**) in MeOH-*d*4. The spectrum was acquired over 4 scans using the standard zg30 Bruker pulse sequence....... 18

Figure S19. 151 MHz ^{13}C NMR spectrum of 1''–dehydrocrinemodin bianthrone (**2**) in MeOH-*d*4. The spectrum was acquired over 10000 scans using the standard zgpg30 Bruker pulse sequence....... 19

Figure S20. ^{1}H–^{13}C HSQC NMR spectrum of 1''–dehydrocrinemodin bianthrone (**2**) in MeOH-d_4. The spectrum was acquired over 64 scans at 1024 t1 increments using the standard hsqcedetgpsisp2.3 Bruker pulse sequence. 20

Figure S21. ^{1}H–^{1}H COSY NMR spectrum of 1''–dehydrocrinemodin bianthrone (**2**) in MeOH-d_4. The spectrum was acquired over 4 scans at 1024 t1 increments using the standard cosygpppqf Bruker pulse sequence. 21

Figure S22. ^{1}H–^{13}C HMBC NMR spectrum of 1''–dehydrocrinemodin bianthrone (**2**) in MeOH-d_4. The spectrum was acquired over 296 scans at 2048 t1 increments using the standard hmbcetgpl3nd Bruker pulse sequence. 22

Figure S23. ^{1}H–^{13}C LRHSQMBC NMR spectrum of 1''–dehydrocrinemodin bianthrone (**2**) in MeOH-d_4. The spectrum was acquired over 300 scans at 2048 t1 increments using the js_lrhsqmbc_final pulse sequence 23

Figure S24. FTIR spectrum for 1''–dehydrocrinemodin bianthrone (**2**). 24

Figure S25. HRESIMS spectrum of 1''–hydroxycrinemodin bianthrone (**3**). 25

Figure S26. Expansion of HRESIMS spectrum of 1''–hydroxycrinemodin bianthrone (**3**). 25

Figure S27. 600 MHz ^{1}H NMR spectrum of 1''–hydroxycrinemodin bianthrone (**3**) in MeOH-d_4. The spectrum was acquired over 4 scans using the standard zg30 Bruker pulse sequence 26

Figure S28. Expansion of 600 MHz ^{1}H NMR spectrum of 1''–hydroxycrinemodin bianthrone (**3**) in MeOH-d_4. The spectrum was acquired over 4 scans using the standard zg30 Bruker pulse sequence. 27

Figure S29. Expansion of 600 MHz ^{1}H NMR spectrum of 1''–hydroxycrinemodin bianthrone (**3**) in MeOH-d_4. The spectrum was acquired over 4 scans using the standard zg30 Bruker pulse sequence. 28

Figure S30. Expansion of 600 MHz ^{1}H NMR spectrum of 1''–hydroxycrinemodin bianthrone (**3**) in MeOH-d_4. The spectrum was acquired over 4 scans using the standard zg30 Bruker pulse sequence. 29

Figure S31. 151 MHz ^{13}C NMR spectrum of 1''–hydroxycrinemodin bianthrone (**3**) in MeOH-d_4. The spectrum was acquired over 12000 scans using the standard zgpg30 Bruker pulse sequence 30

Figure S32. ^{1}H–^{1}H COSY NMR spectrum of 1''–hydroxycrinemodin bianthrone (**3**) in MeOH-*d*$_4$. The spectrum was acquired over 4 scans at 1024 t1 increments using the standard cosygpppqf Bruker pulse sequence. 31
Figure S33. ^{1}H–^{13}C HSQC NMR spectrum of 1''–hydroxycrinemodin bianthrone (**3**) in MeOH-*d*$_4$. The spectrum was acquired over 64 scans at 1024 t1 increments using the standard hsqcedetgpsisp2.3 Bruker pulse sequence. 32
Figure S34. ^{1}H–^{13}C HMBC NMR spectrum of 1''–hydroxycrinemodin bianthrone (**3**) in MeOH-*d*$_4$. The spectrum was acquired over 240 scans at 2048 t1 increments using the standard hmbcetgpl3nd Bruker pulse sequence. 33
Figure S35. FTIR spectrum of 1''–hydroxycrinemodin bianthrone (**3**). 34
Figure S36. ECD spectra overlap of compounds **1**–**3** in acetonitrile. 34
Figure S37. ^{1}H NMR spectra of **3** (in MeOH-*d*$_4$) titration with D_2O, from 0 μL (bottom) to 20 μL (top) of D_2O. 35
Figure S38. NCI60 data for crinemodin bianthrone (**1**). 36
Figure S39. NCI60 data for 1''–dehydrocrinemodin bianthrone (**2**). 37
Figure S40. NCI60 data for 1''–hydroxycrinemodin bianthrone (**3**). 38

LIST OF TABLES

Table S1. Reaction rates of **3** reacted with propionic anhydride using *R*- or *S*-HBTM catalysts. The reaction rate was achieved by measuring the peak areas for the fully acylated derivative, using the extracted ion chromatogram (EIC) for the sodiated molecule $[M + Na]^+$ at *m/z* 997.3617 (± 20 ppm). 39

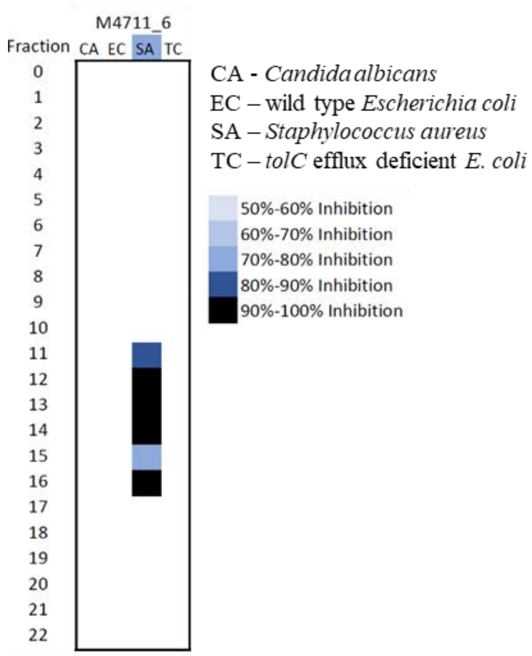

Figure S1. Screening of subfractions generatedfor the an antimicrobial campaing between NIAID and NCI.[1]

1. L. Martinez-Fructuoso, S. J. R. Arends, V. F. Freire, J. R. Evans, S. DeVries, B. D. Peyser, R. K. Akee, C. C. Thornburg, R. Kumar, S. Ensel, G. M. Morgan, G. D. McConachie, N. Veeder, L. R. Duncan, T. Grkovic and B. R. O'Keefe, *ACS Infect. Dis.*, 2023, **9**, 1245-1256.

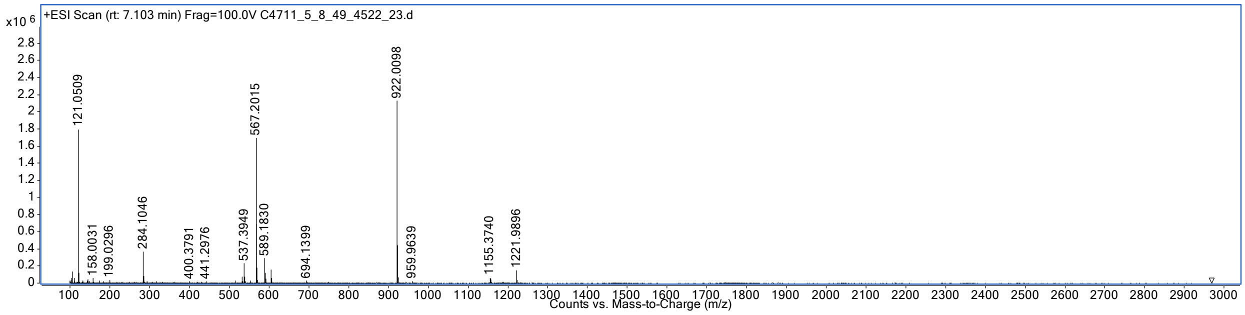

Figure S2. HRESIMS spectrum of crinemodin bianthrone (**1**).

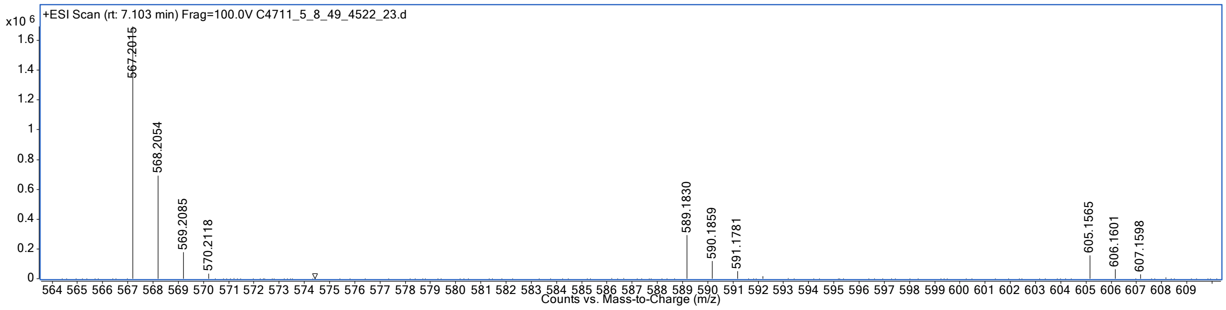

Figure S3. Expansion of HRESIMS spectrum of crinemodin bianthrone (**1**).

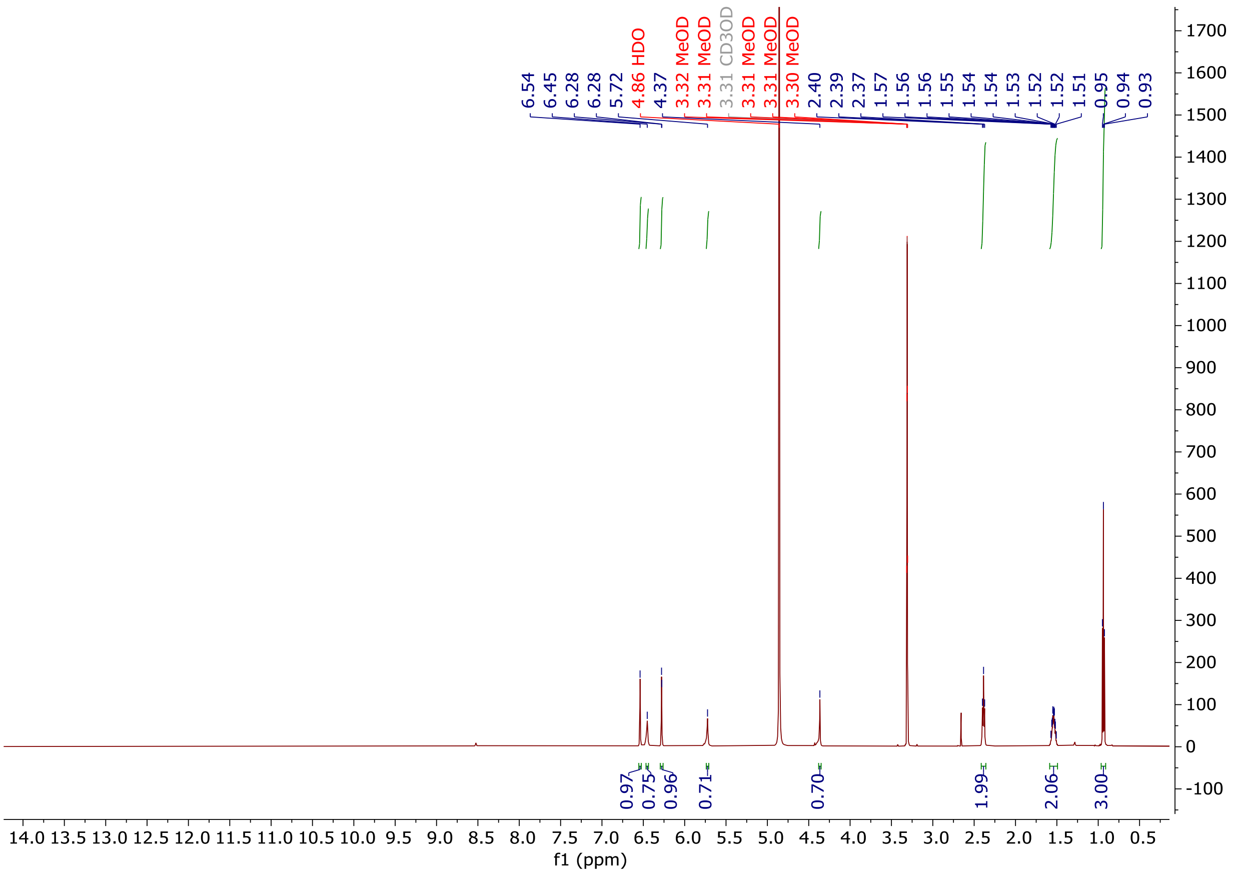

Figure S4. 600 MHz ^{1}H NMR spectrum of crinemodin bianthrone (**1**) in MeOH-d_4. The spectrum was acquired over 4 scans using the standard zg30 Bruker pulse sequence.

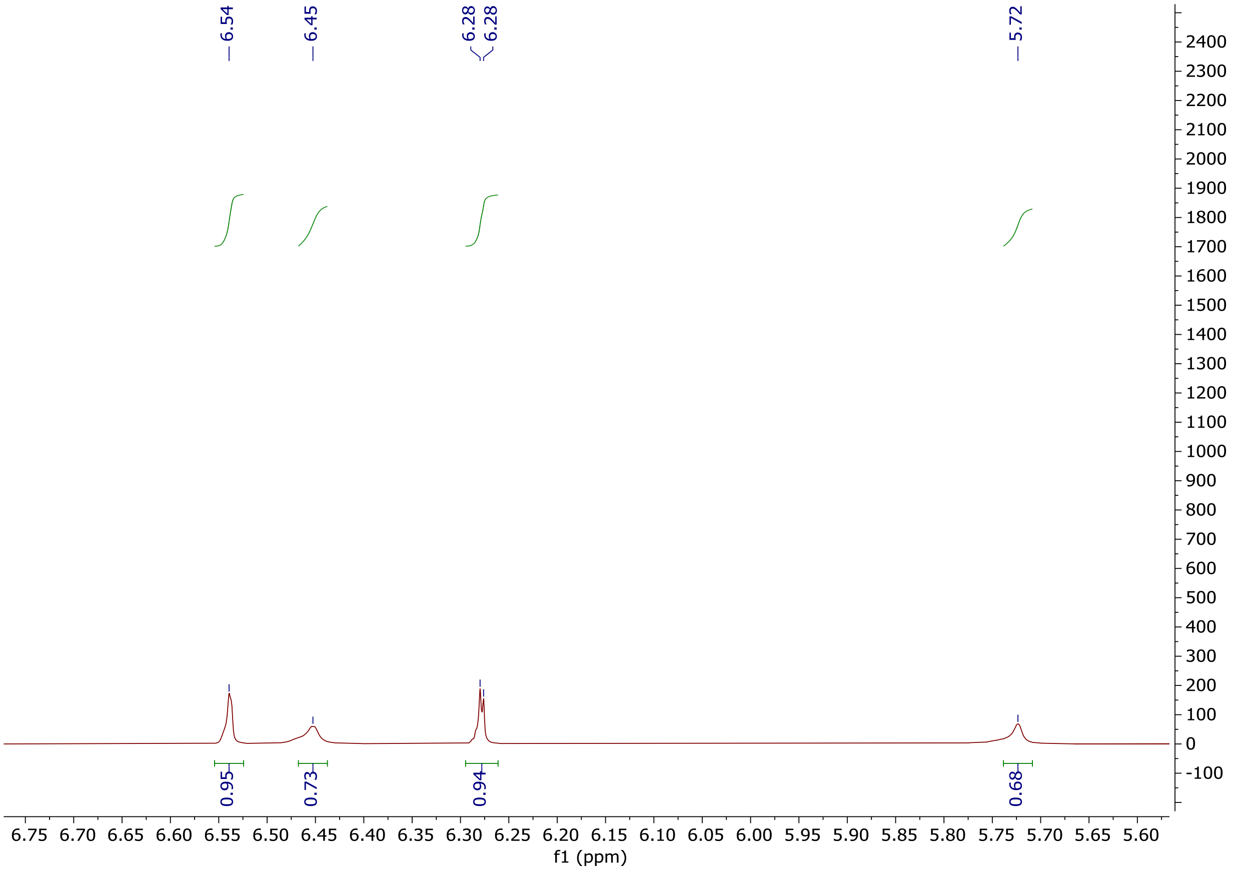

Figure S5. Expansion of 600 MHz ^{1}H NMR spectrum of crinemodin bianthrone (**1**) in MeOH-d_4. The spectrum was acquired over 4 scans using the standard zg30 Bruker pulse sequence.

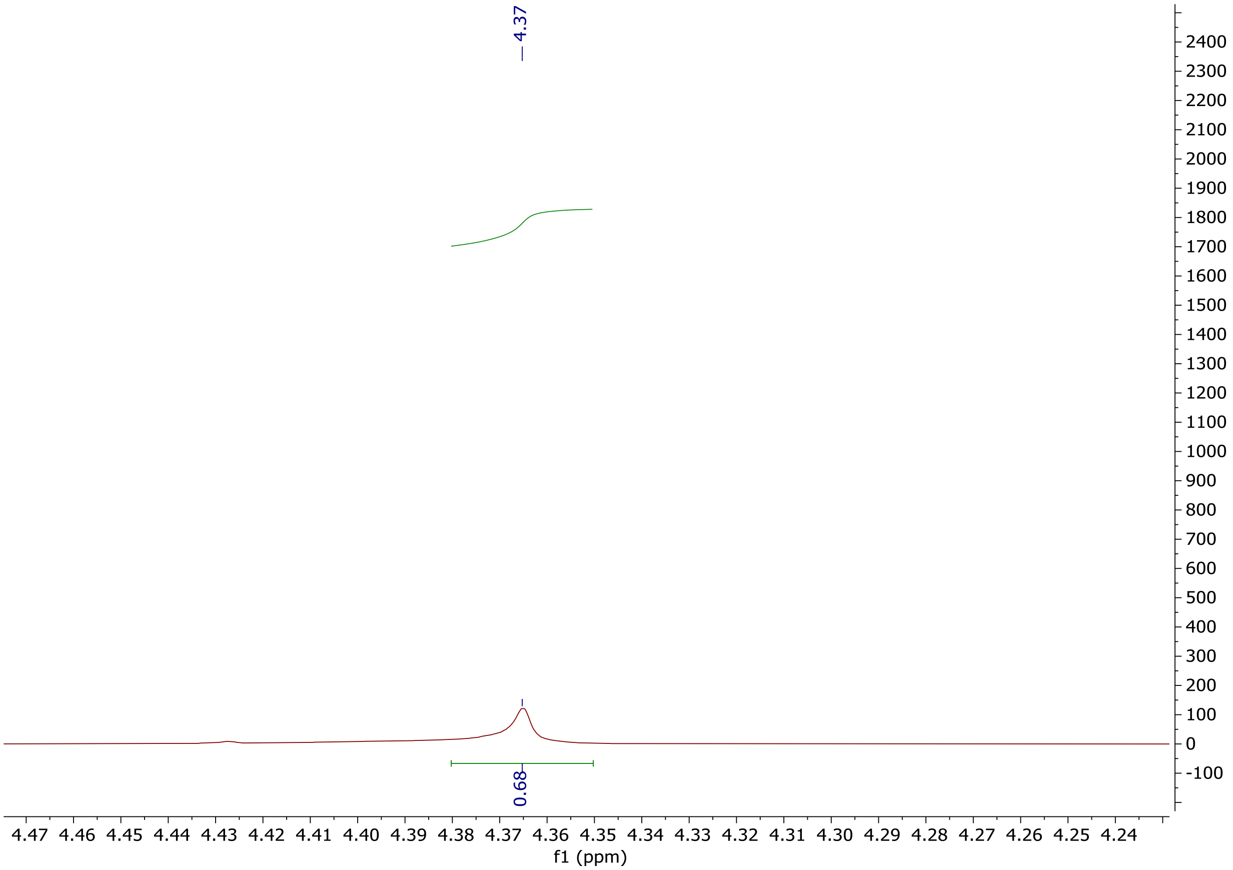

Figure S6. Expansion of 600 MHz ^{1}H NMR spectrum of crinemodin bianthrone (**1**) in MeOH-d_4. The spectrum was acquired over 4 scans using the standard zg30 Bruker pulse sequence.

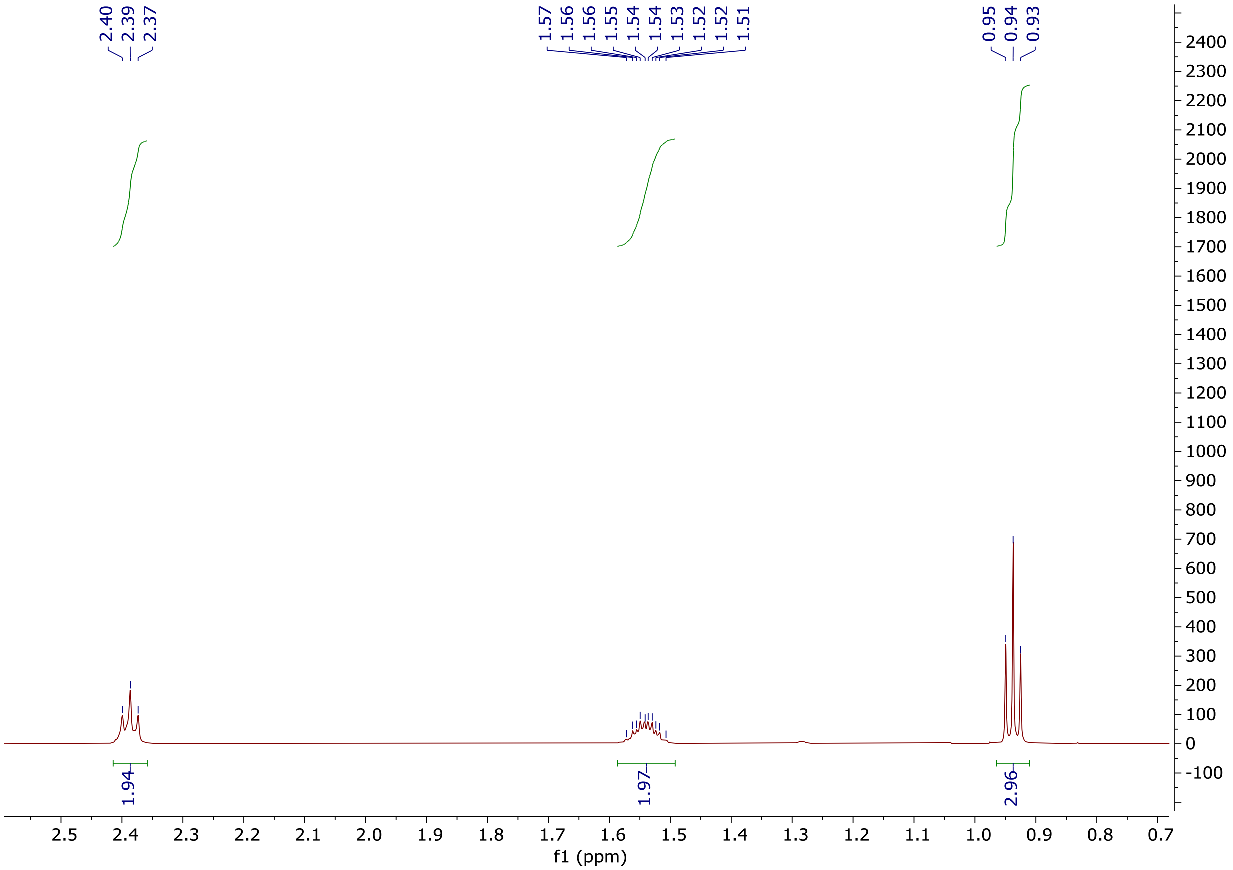

Figure S7. Expansion of 600 MHz ^{1}H NMR spectrum of crinemodin bianthrone (**1**) in MeOH-*d*$_4$. The spectrum was acquired over 4 scans using the standard zg30 Bruker pulse sequence.

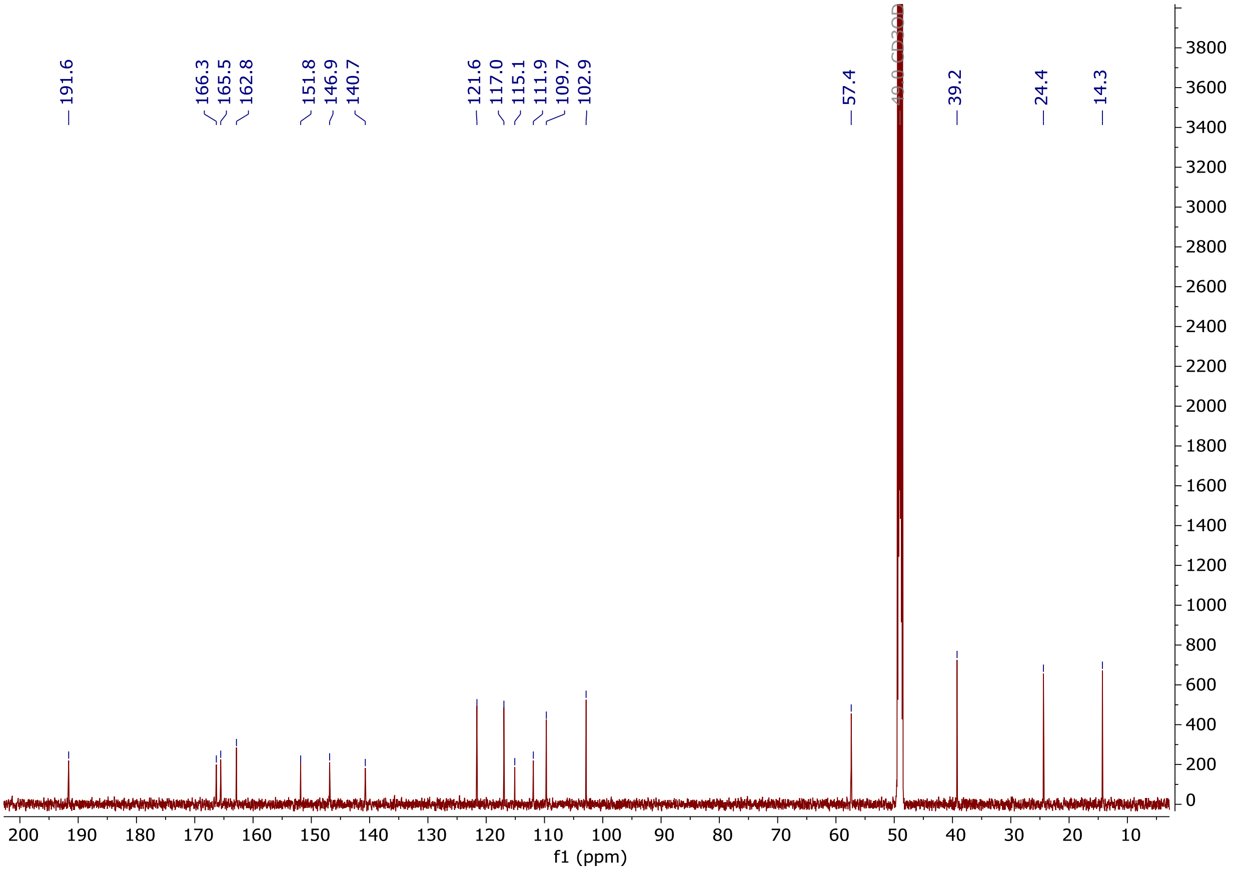

Figure S8. 151 MHz ^{13}C NMR spectrum of crinemodin bianthrone (**1**) in MeOH-*d*$_4$. The spectrum was acquired over 10000 scans using the standard zgpg30 Bruker pulse sequence.

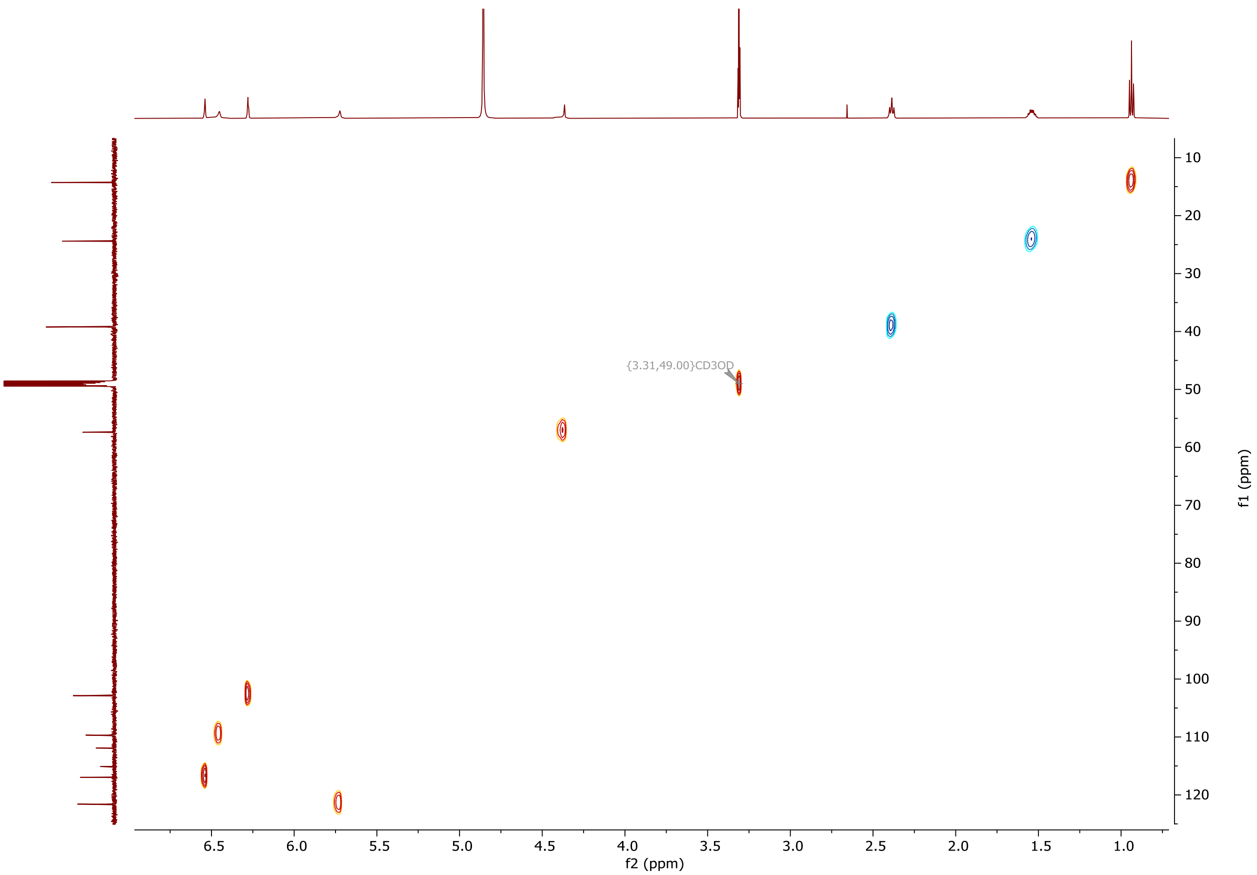

Figure S9. ^{1}H–^{13}C HSQC NMR spectrum of crinemodin bianthrone (**1**) in MeOH-*d*$_4$. The spectrum was acquired over 80 scans at 1024 t1 increments using the standard hsqcedetgpsisp2.3 Bruker pulse sequence.

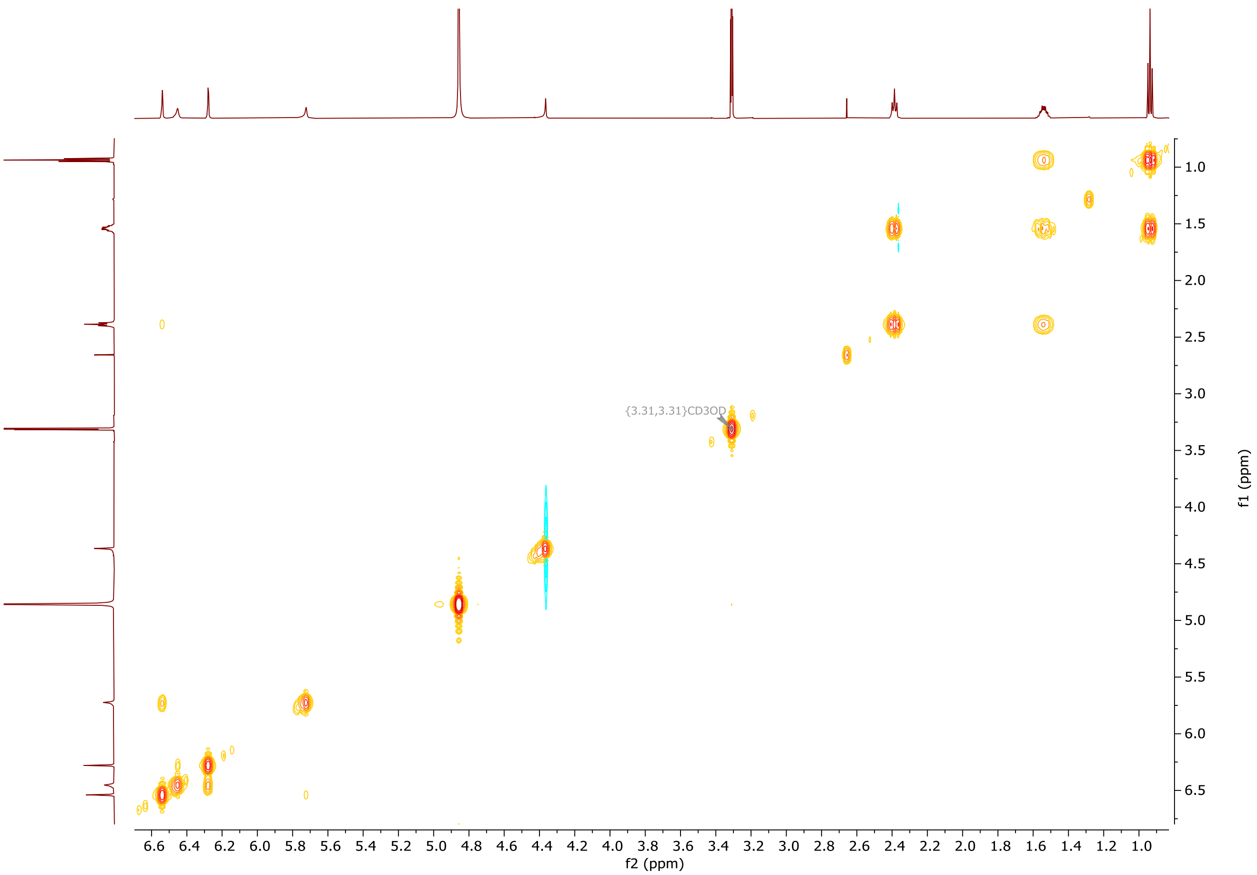

Figure S10. ^{1}H–^{1}H COSY NMR spectrum of crinemodin bianthrone (**1**) in MeOH-*d*$_4$. The spectrum was acquired over 8 scans at 1024 t1 increments using the standard cosygpppqf Bruker pulse sequence.

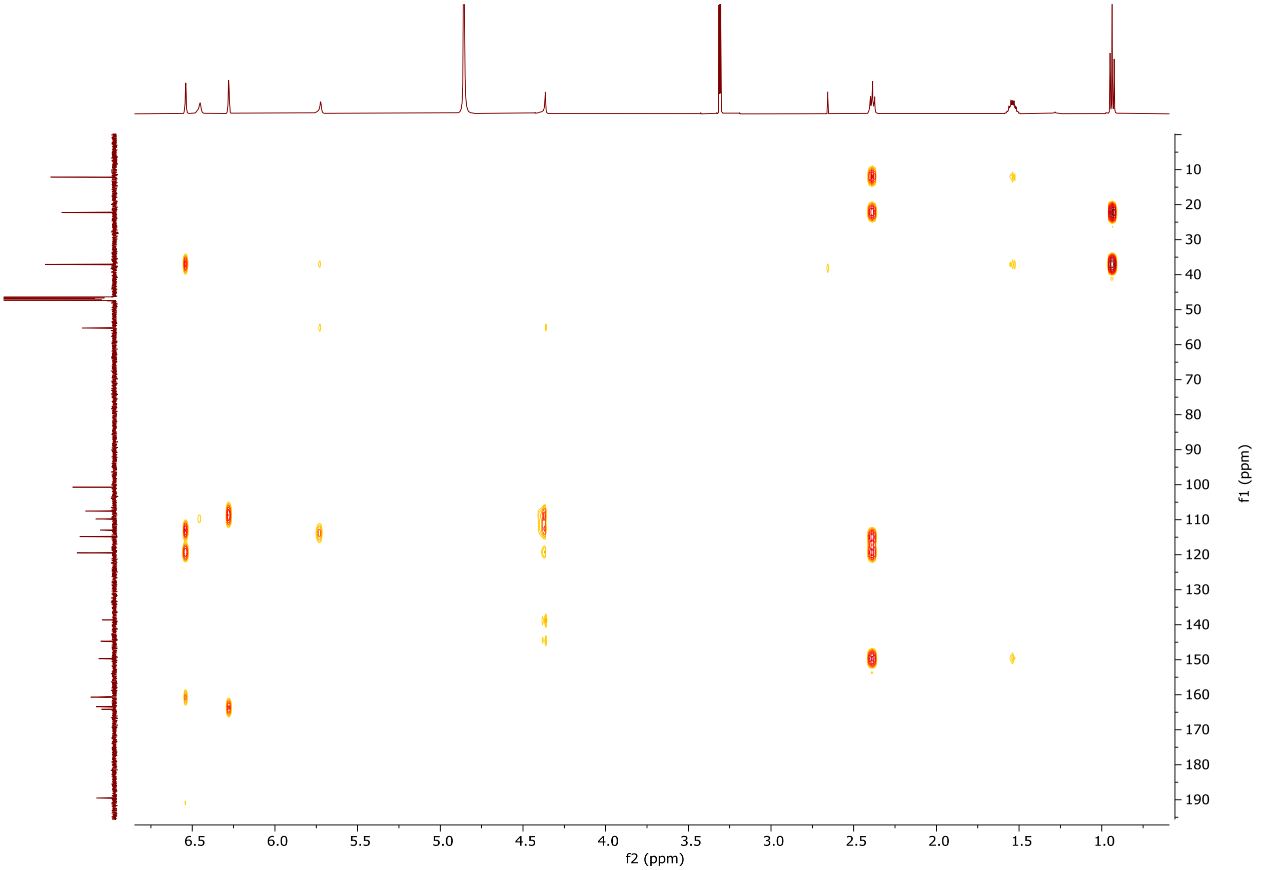

Figure S11. ^{1}H–^{13}C HMBC NMR spectrum of crinemodin bianthrone (**1**) in MeOH-d_4. The spectrum was acquired over 296 scans at 2048 t1 increments using the standard hmbcetgpl3nd Bruker pulse sequence.

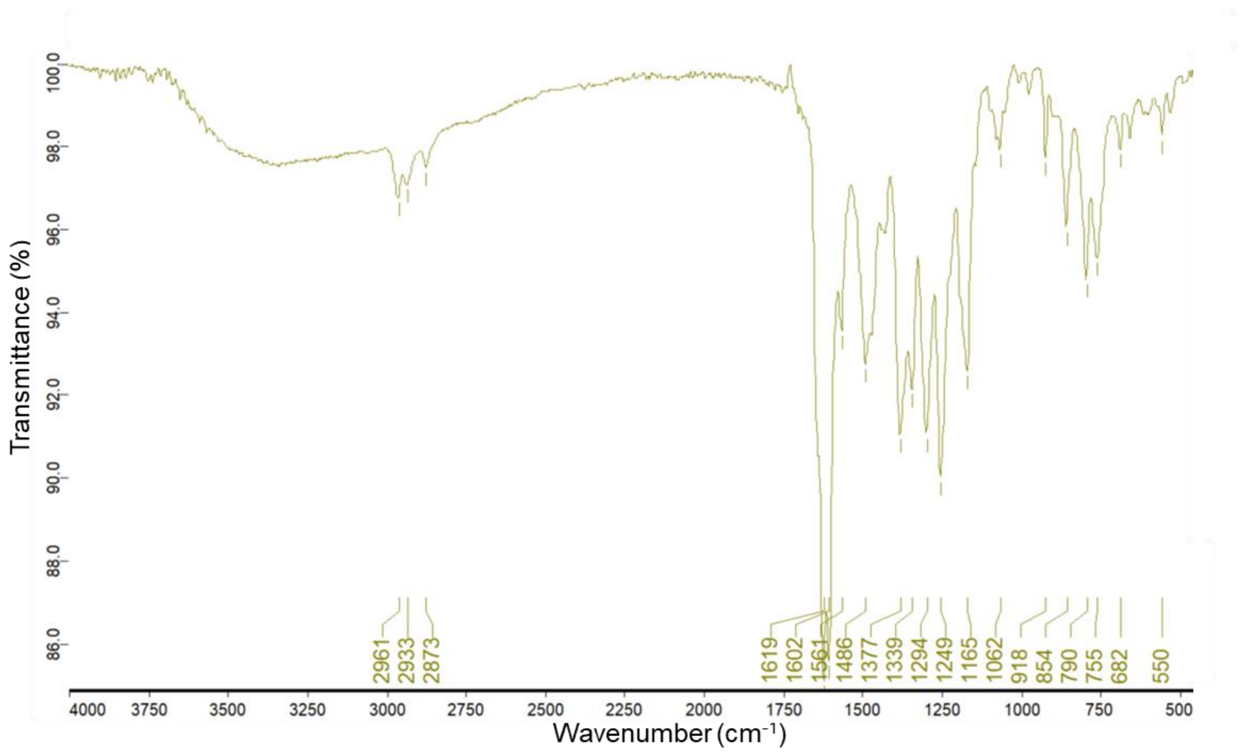

Figure S12. FTIR spectrum of crinemodin bianthrone (**1**).

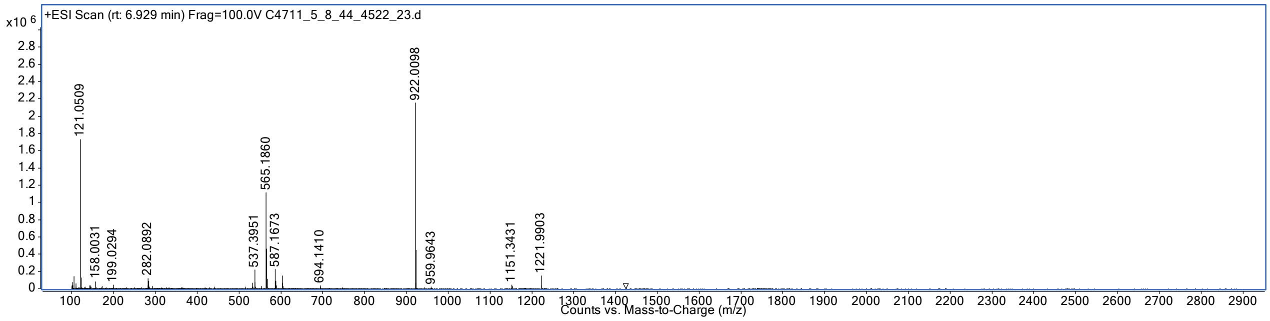

Figure S13. HRESIMS spectrum of 1"–dehydrocrinemodin bianthrone (**2**).

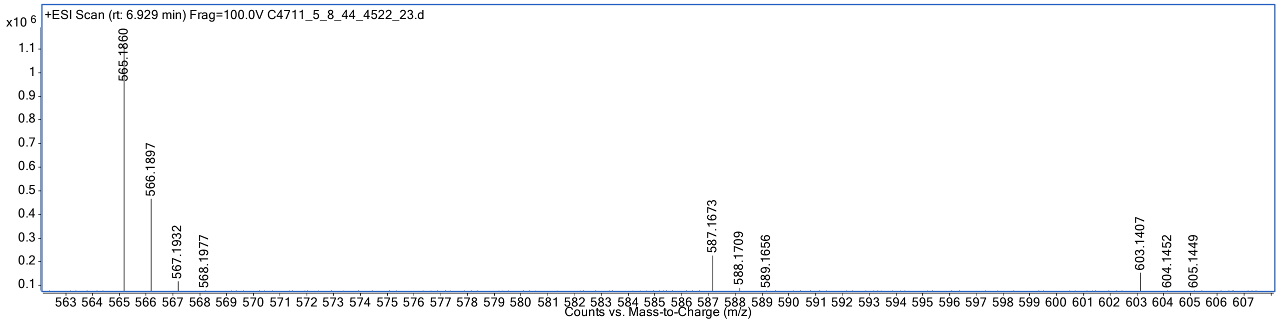

Figure S14. Expansion of HRESIMS spectrum of 1"–dehydrocrinemodin bianthrone (**2**).

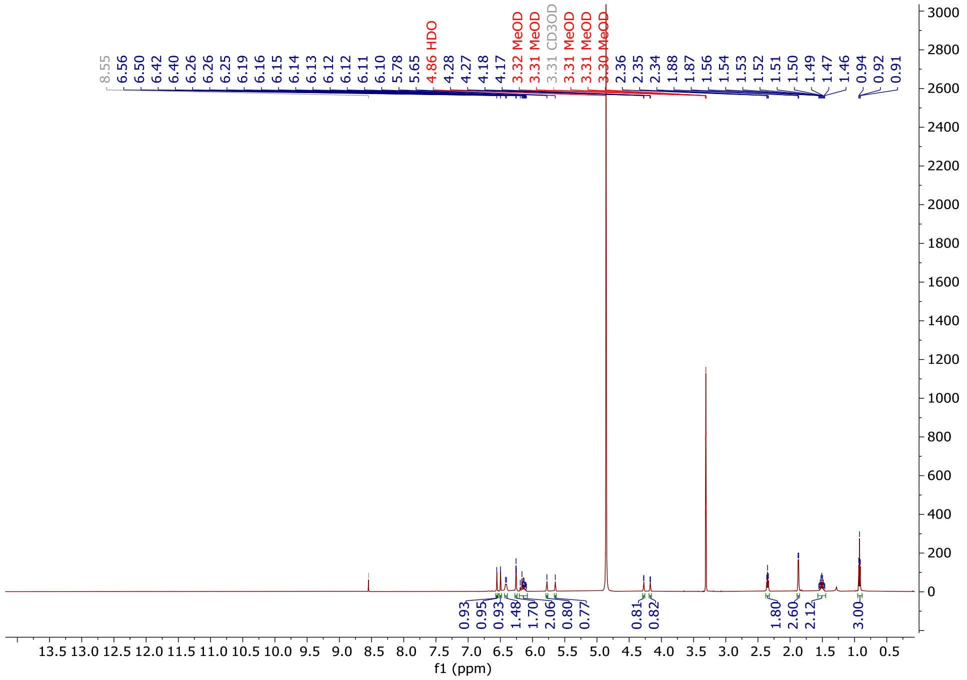

Figure S15. 600 MHz ^{1}H NMR spectrum of 1"–dehydrocrinemodin bianthrone (**2**) in MeOH-d_4. The spectrum was acquired over 4 scans using the standard zg30 Bruker pulse sequence.

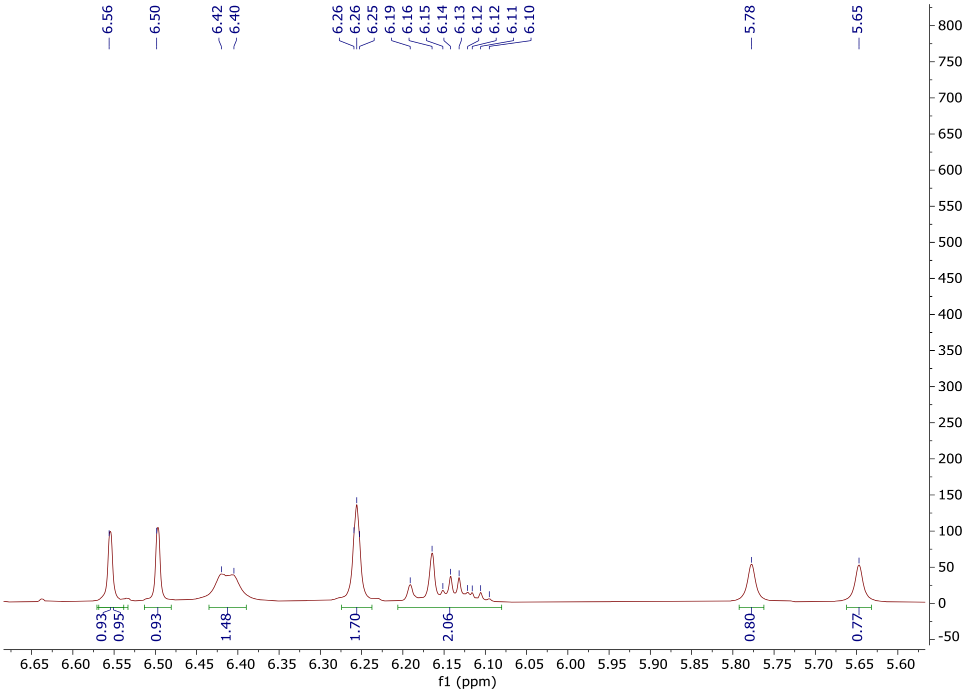

Figure S16. Expansion of 600 MHz ^{1}H NMR spectrum of 1''–dehydrocrinemodin bianthrone (**2**) in MeOH-d_4. The spectrum was acquired over 4 scans using the standard zg30 Bruker pulse sequence.

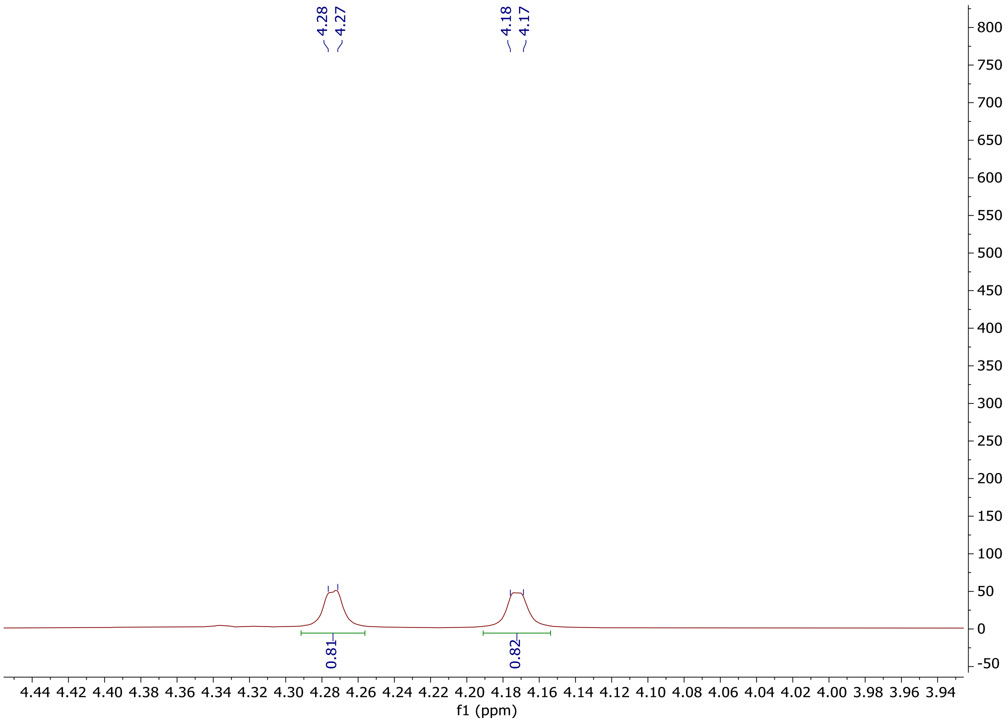

Figure S17. Expansion of 600 MHz ^{1}H NMR spectrum of 1''–dehydrocrinemodin bianthrone (**2**) in MeOH-d_4. The spectrum was acquired over 4 scans using the standard zg30 Bruker pulse sequence.

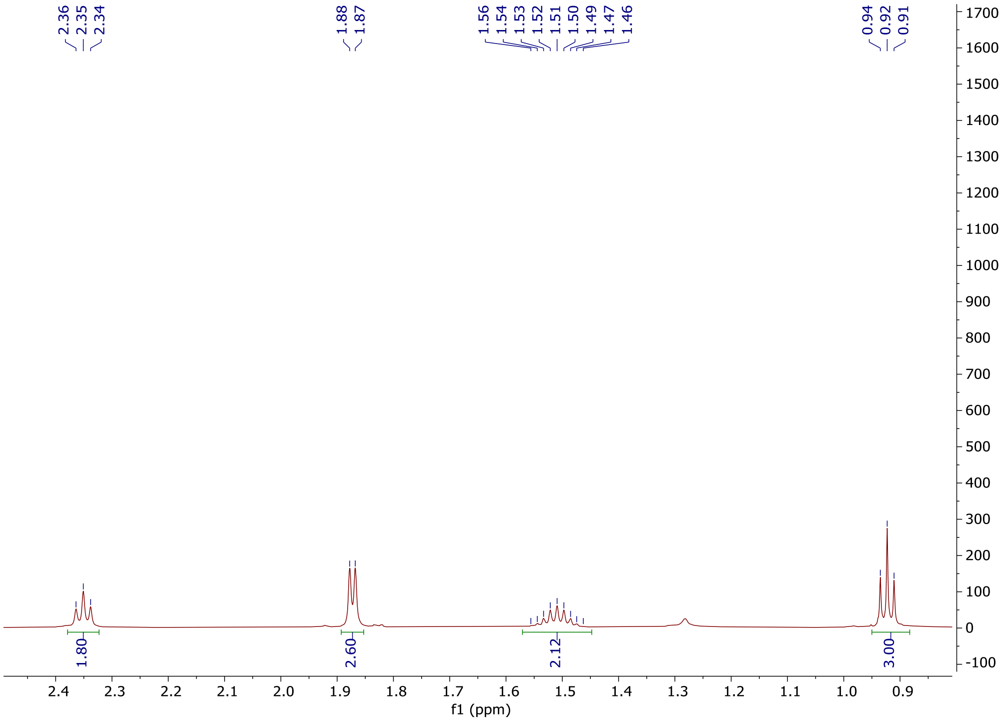

Figure S18. Expansion of 600 MHz ^{1}H NMR spectrum of 1''–dehydrocrinemodin bianthrone (**2**) in MeOH-d_4. The spectrum was acquired over 4 scans using the standard zg30 Bruker pulse sequence.

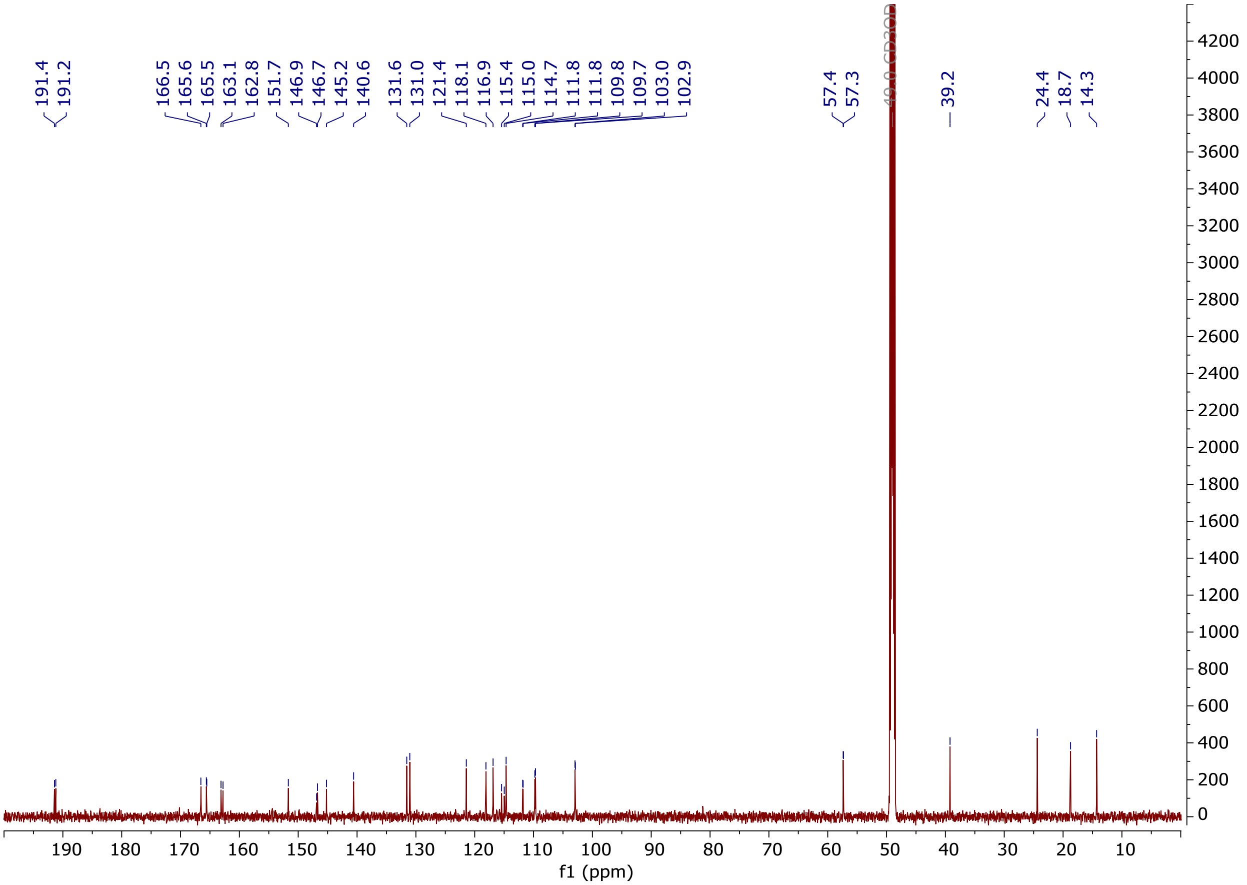

Figure S19. 151 MHz ^{13}C NMR spectrum of 1''–dehydrocrinemodin bianthrone (**2**) in MeOH-*d*4. The spectrum was acquired over 10000 scans using the standard zgpg30 Bruker pulse sequence.

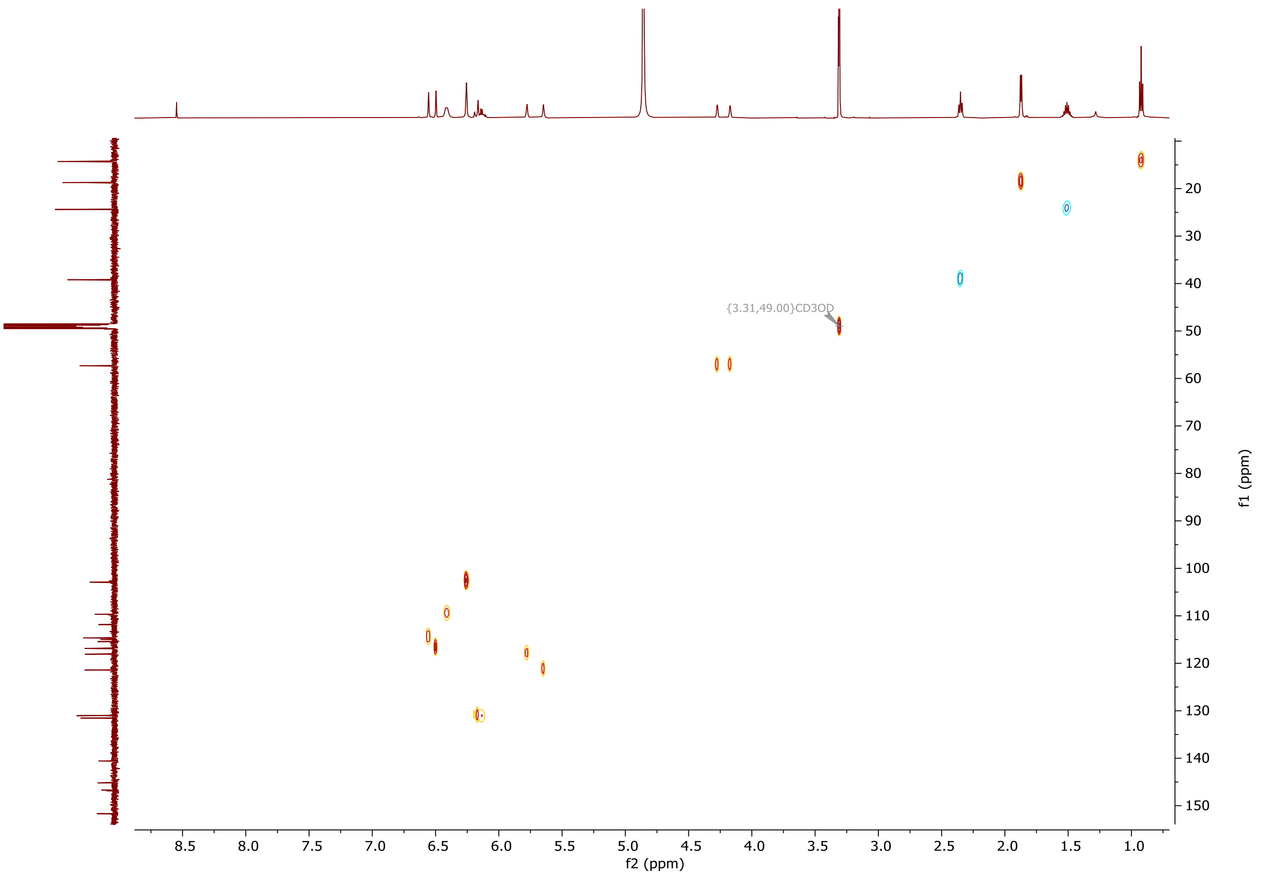

Figure S20. ^{1}H–^{13}C HSQC NMR spectrum of 1''–dehydrocrinemodin bianthrone (**2**) in MeOH-*d*$_4$. The spectrum was acquired over 64 scans at 1024 t1 increments using the standard hsqcedetgpsisp2.3 Bruker pulse sequence.

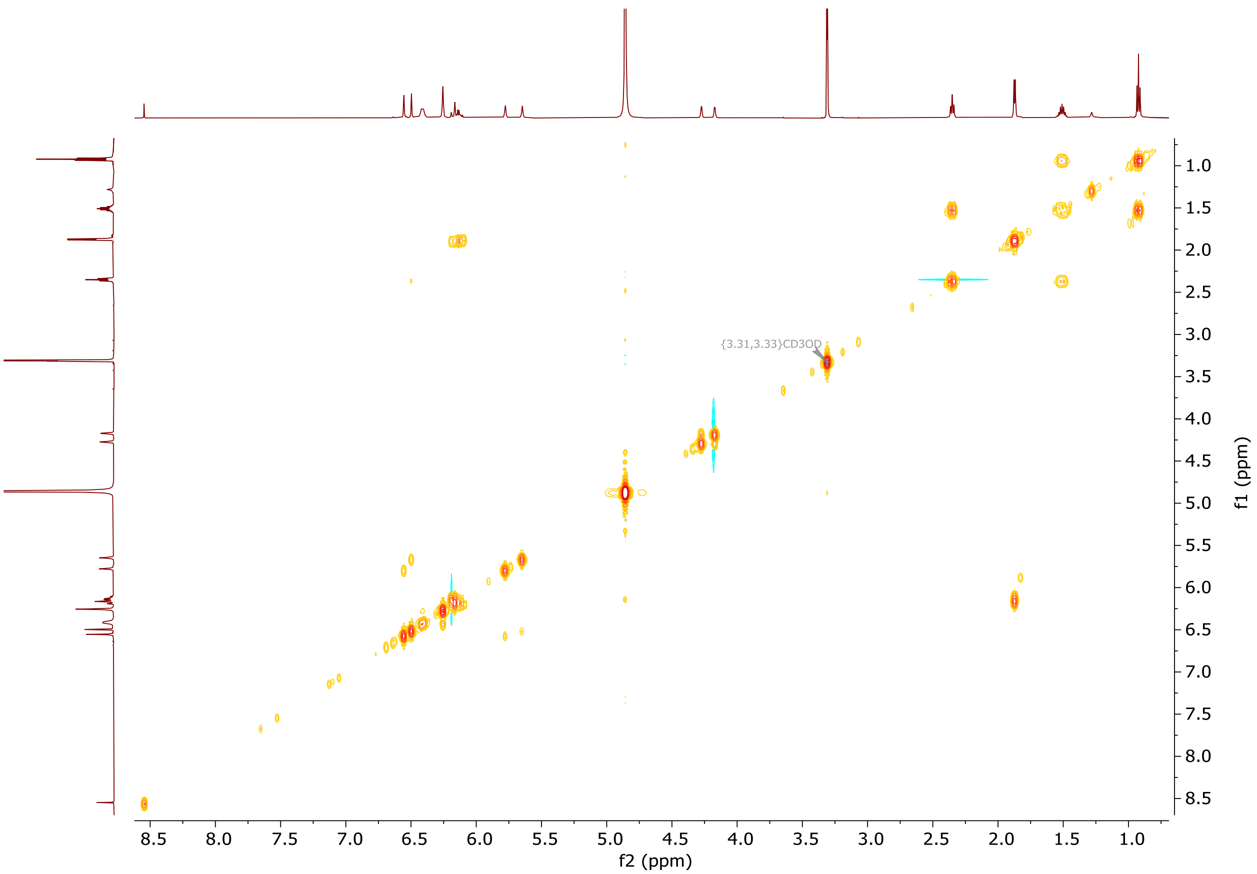

Figure S21. ^{1}H–^{1}H COSY NMR spectrum of 1''–dehydrocrinemodin bianthrone (**2**) in MeOH-*d*4. The spectrum was acquired over 4 scans at 1024 t1 increments using the standard cosygpppqf Bruker pulse sequence.

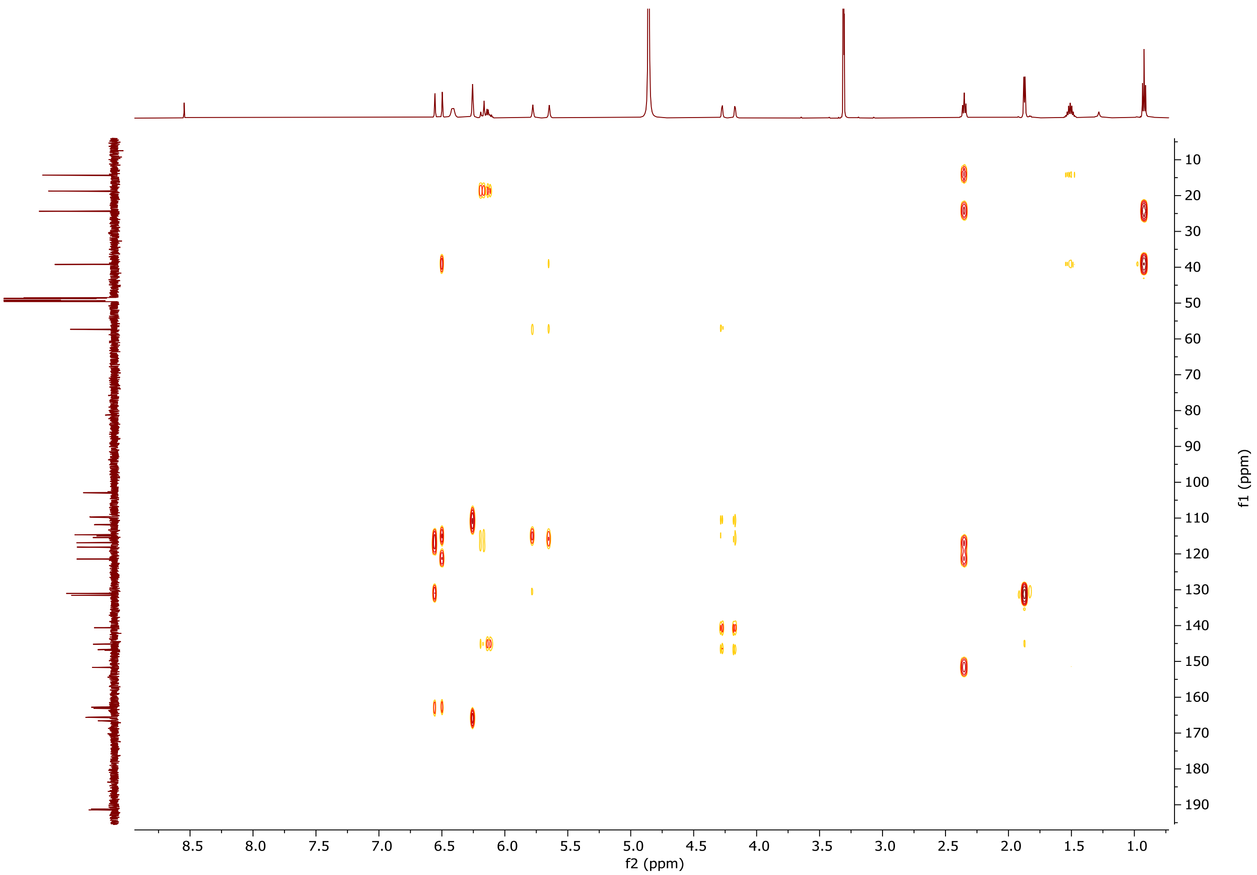

Figure S22. ^{1}H–^{13}C HMBC NMR spectrum of 1''–dehydrocrinemodin bianthrone (**2**) in MeOH-d_4. The spectrum was acquired over 296 scans at 2048 t1 increments using the standard hmbcetgpl3nd Bruker pulse sequence.

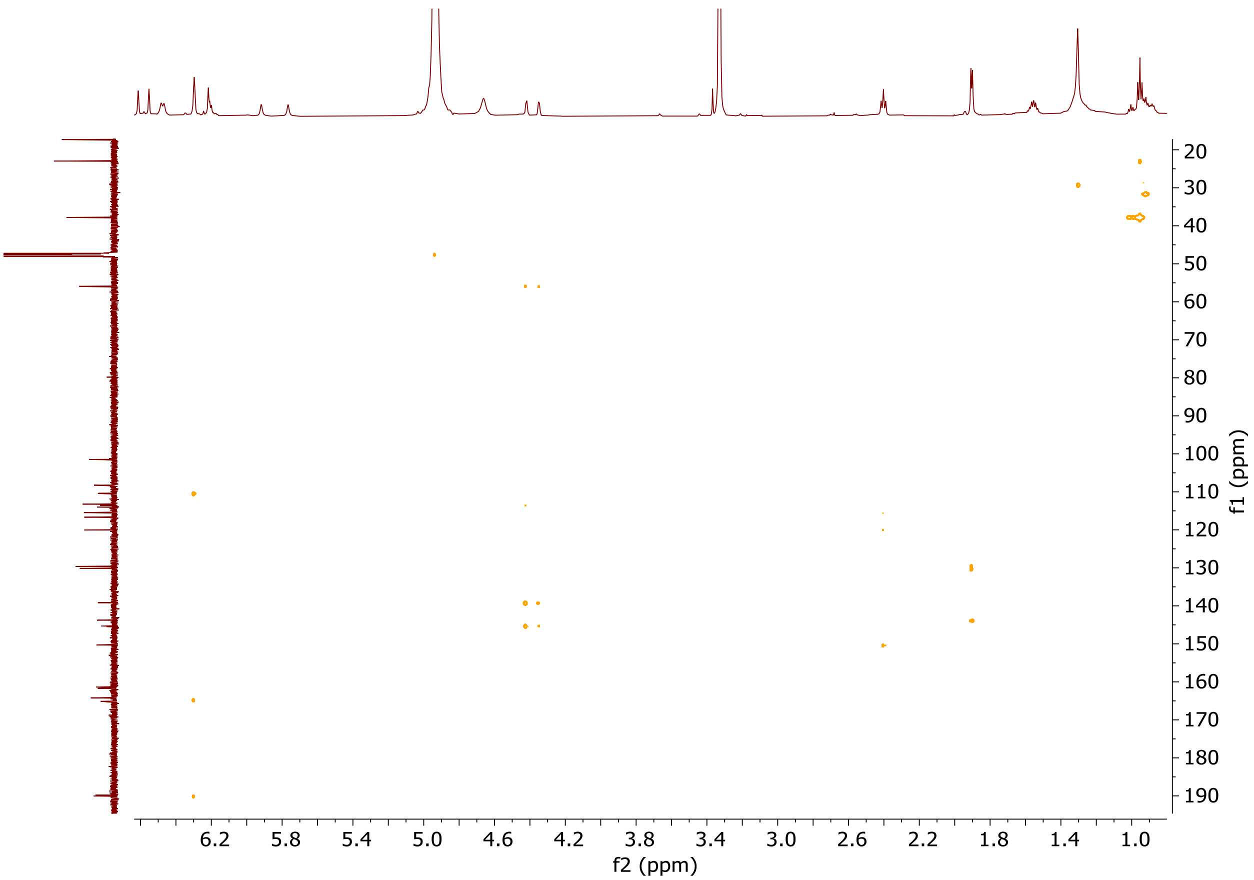

Figure S23. ^{1}H–^{13}C LRHSQMBC NMR spectrum of 1''–dehydrocrinemodin bianthrone (**2**) in MeOH-d_4. The spectrum was acquired over 300 scans at 2048 t1 increments using the js_lrhsqmbc_final pulse sequence.

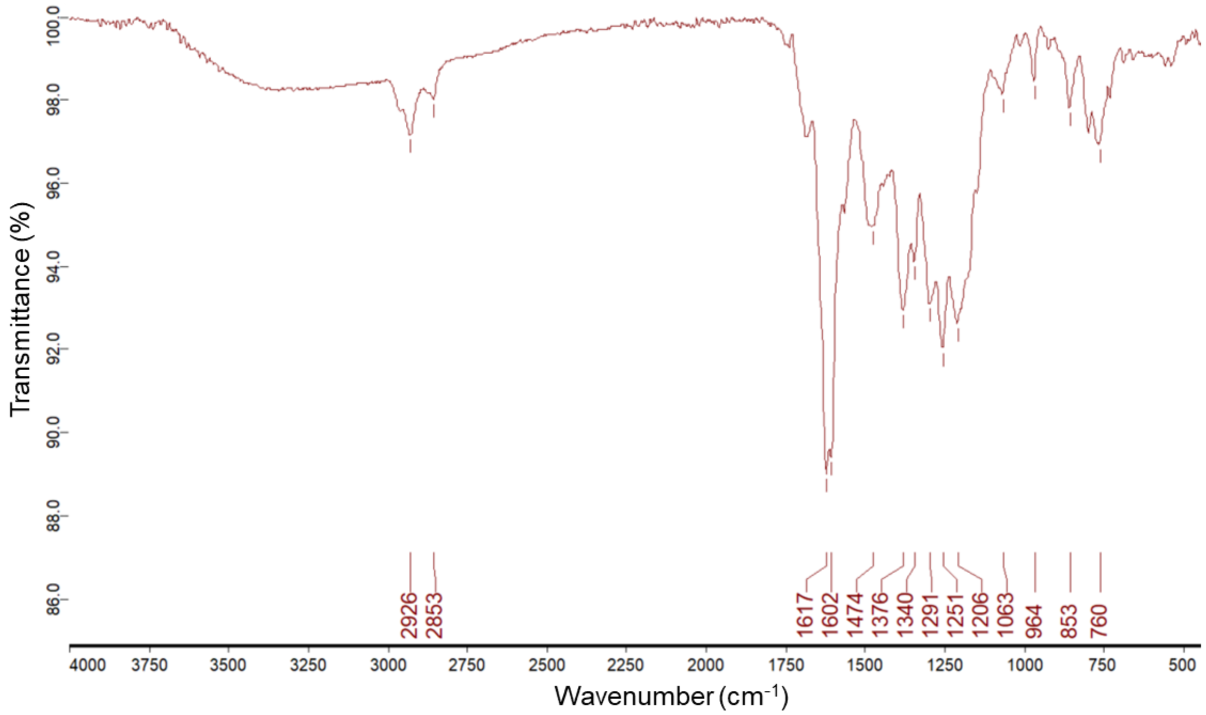

Figure S24. FTIR spectrum for 1''–dehydrocrinemodin bianthrone (**2**).

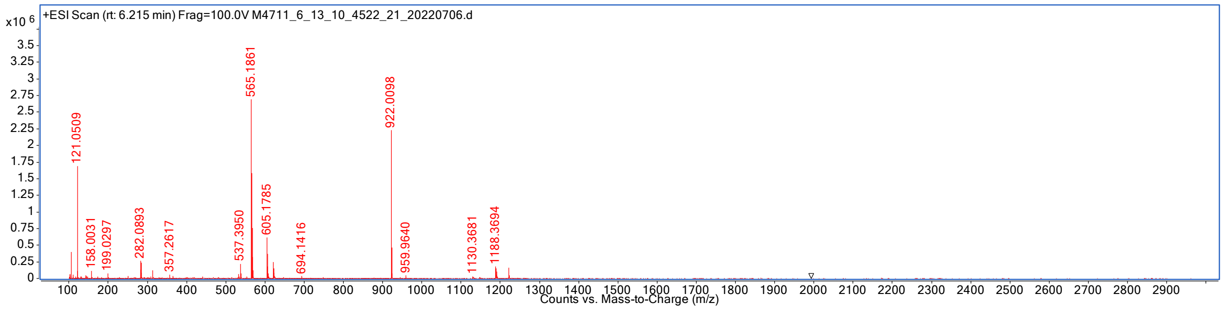

Figure S25. HRESIMS spectrum of 1"–hydroxycrinemodin bianthrone (**3**).

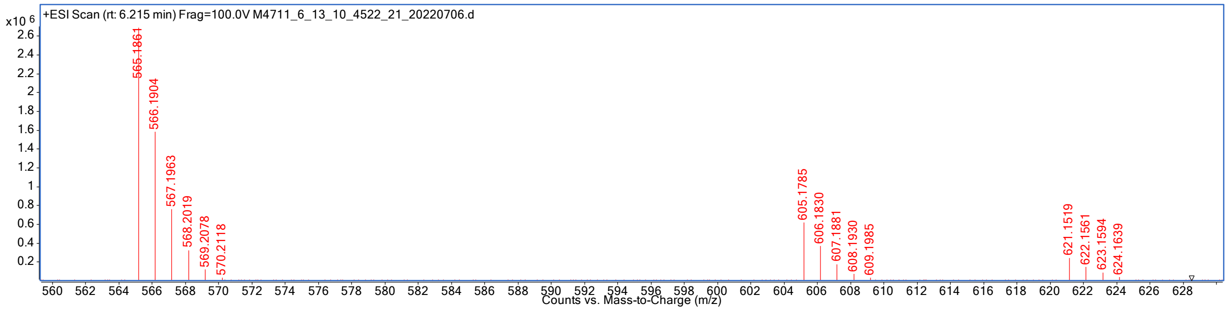

Figure S26. Expansion of HRESIMS spectrum of 1"–hydroxycrinemodin bianthrone (**3**).

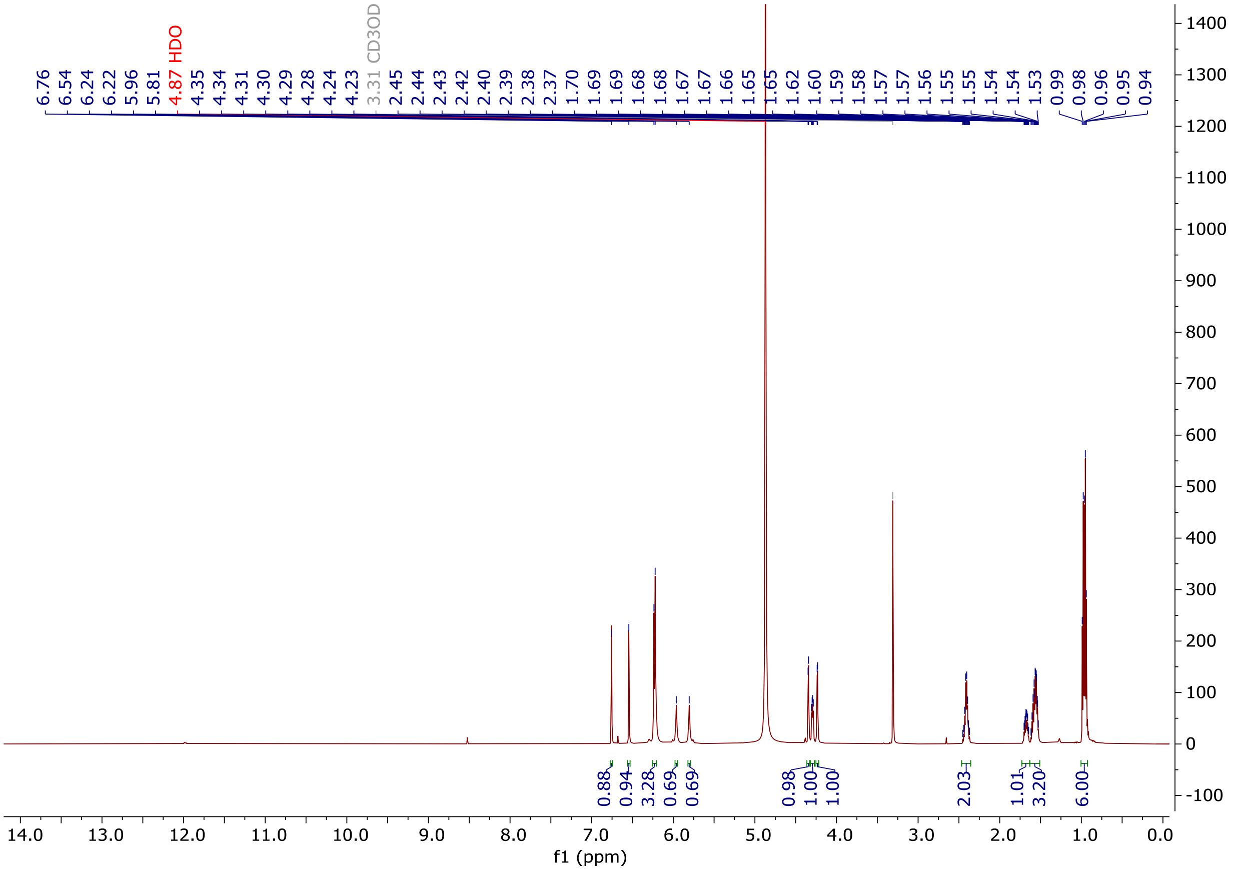

Figure S27. 600 MHz ^{1}H NMR spectrum of 1"–hydroxycrinemodin bianthrone (**3**) in MeOH-d_4. The spectrum was acquired over 4 scans using the standard zg30 Bruker pulse sequence.

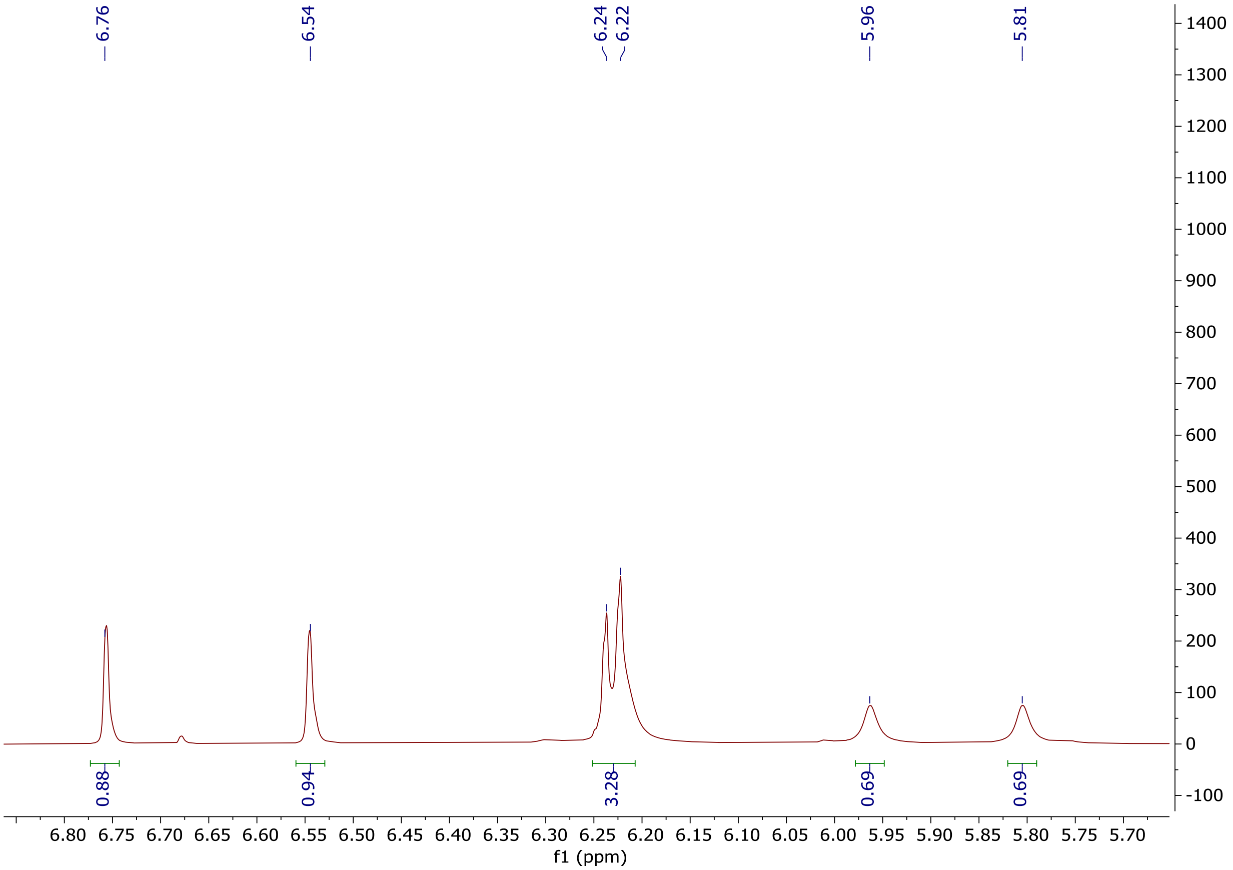

Figure S28. Expansion of 600 MHz ^{1}H NMR spectrum of 1''–hydroxycrinemodin bianthrone (**3**) in MeOH-*d*$_4$. The spectrum was acquired over 4 scans using the standard zg30 Bruker pulse sequence.

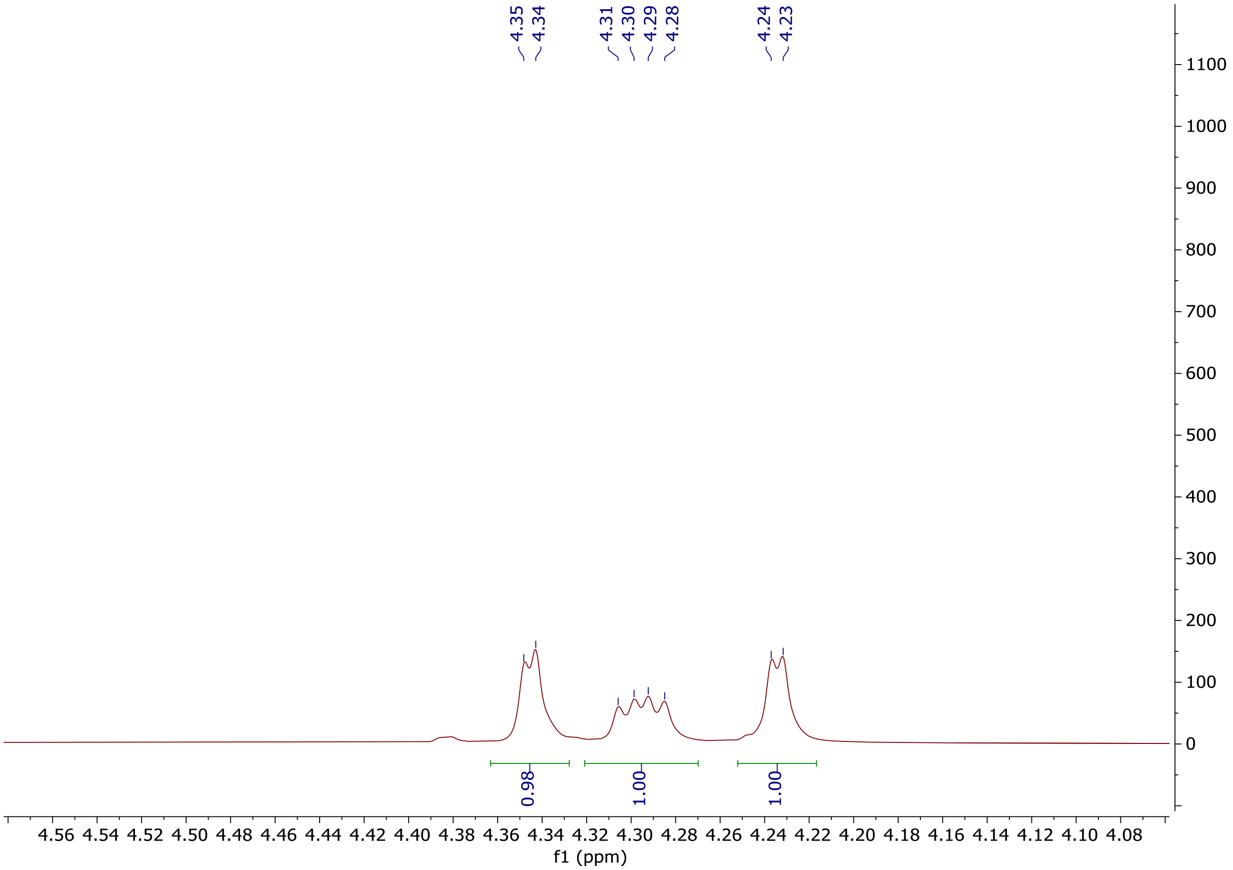

Figure S29. Expansion of 600 MHz ^{1}H NMR spectrum of 1''–hydroxycrinemodin bianthrone (**3**) in MeOH-*d*$_4$. The spectrum was acquired over 4 scans using the standard zg30 Bruker pulse sequence.

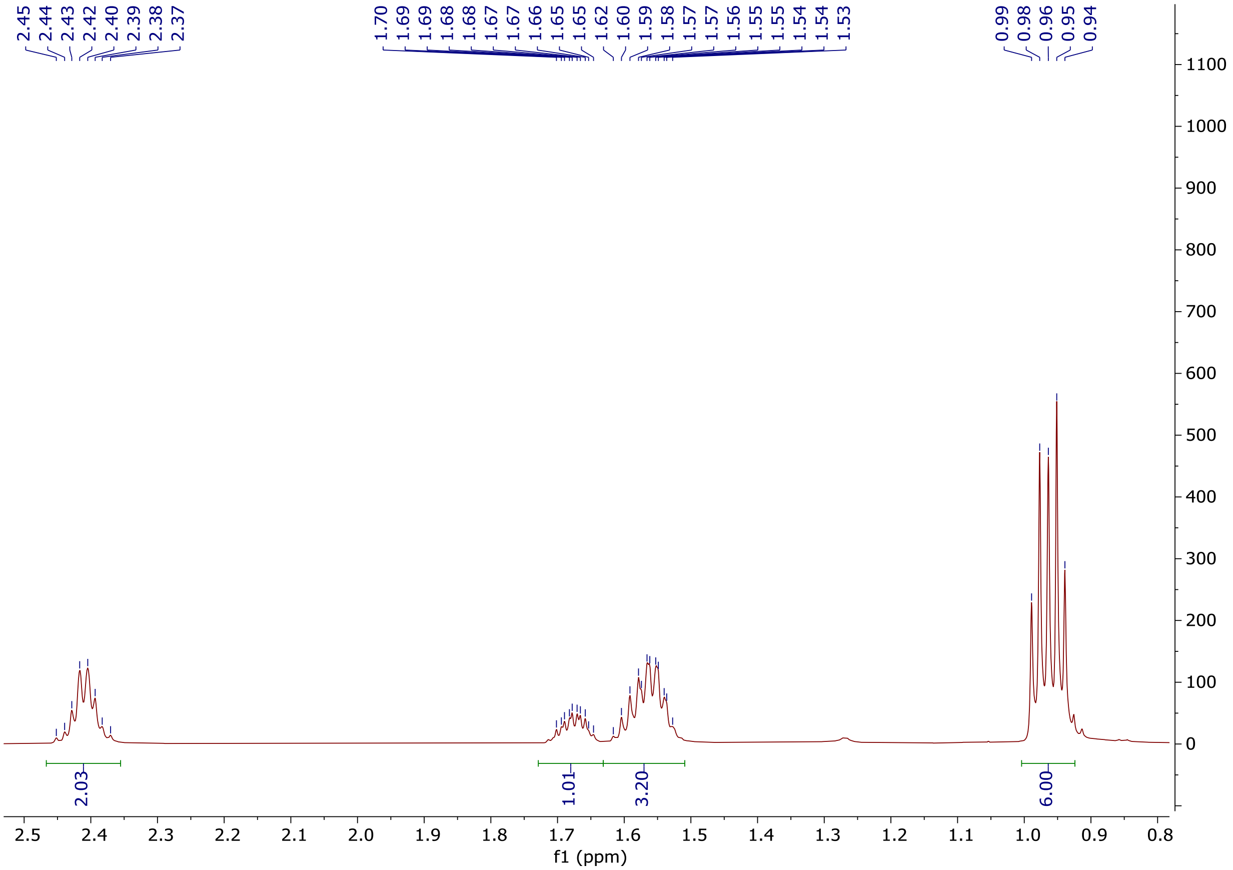

Figure S30. Expansion of 600 MHz ^{1}H NMR spectrum of 1''–hydroxycrinemodin bianthrone (**3**) in MeOH-*d*4. The spectrum was acquired over 4 scans using the standard zg30 Bruker pulse sequence.

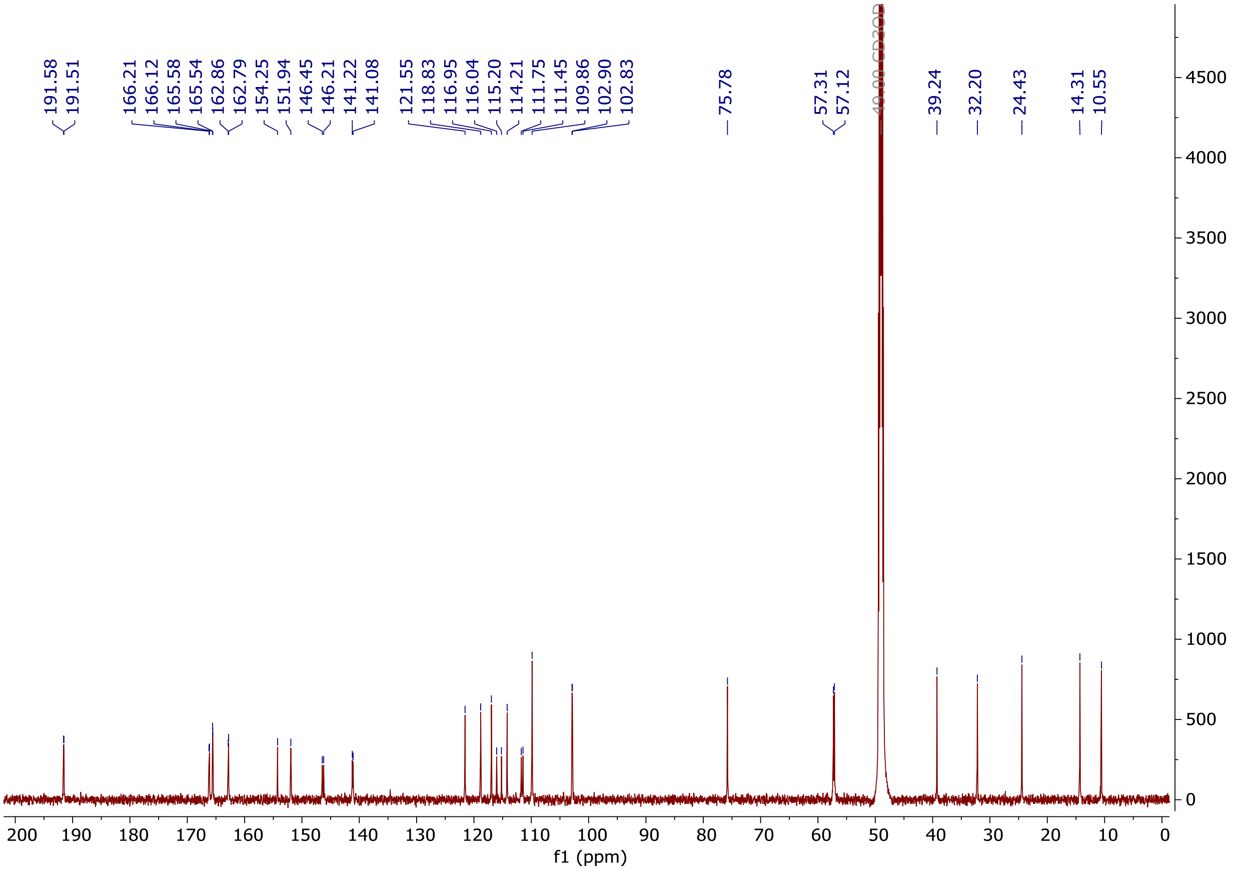

Figure S31. 151 MHz ^{13}C NMR spectrum of 1''–hydroxycrinemodin bianthrone (**3**) in MeOH-*d*$_4$. The spectrum was acquired over 12000 scans using the standard zgpg30 Bruker pulse sequence.

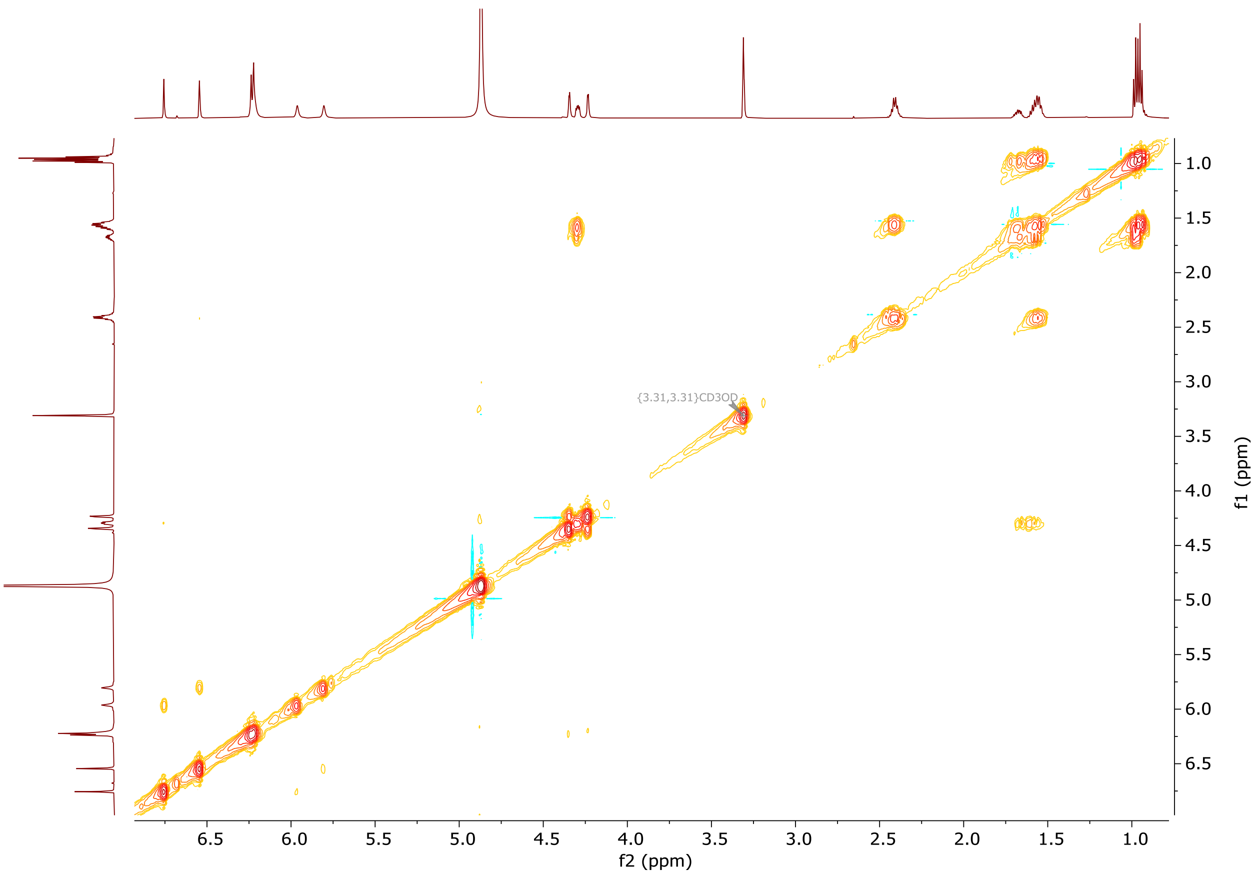

Figure S32. ^{1}H–^{1}H COSY NMR spectrum of 1''–hydroxycrinemodin bianthrone (**3**) in MeOH-d_4. The spectrum was acquired over 4 scans at 1024 t1 increments using the standard cosygpppqf Bruker pulse sequence.

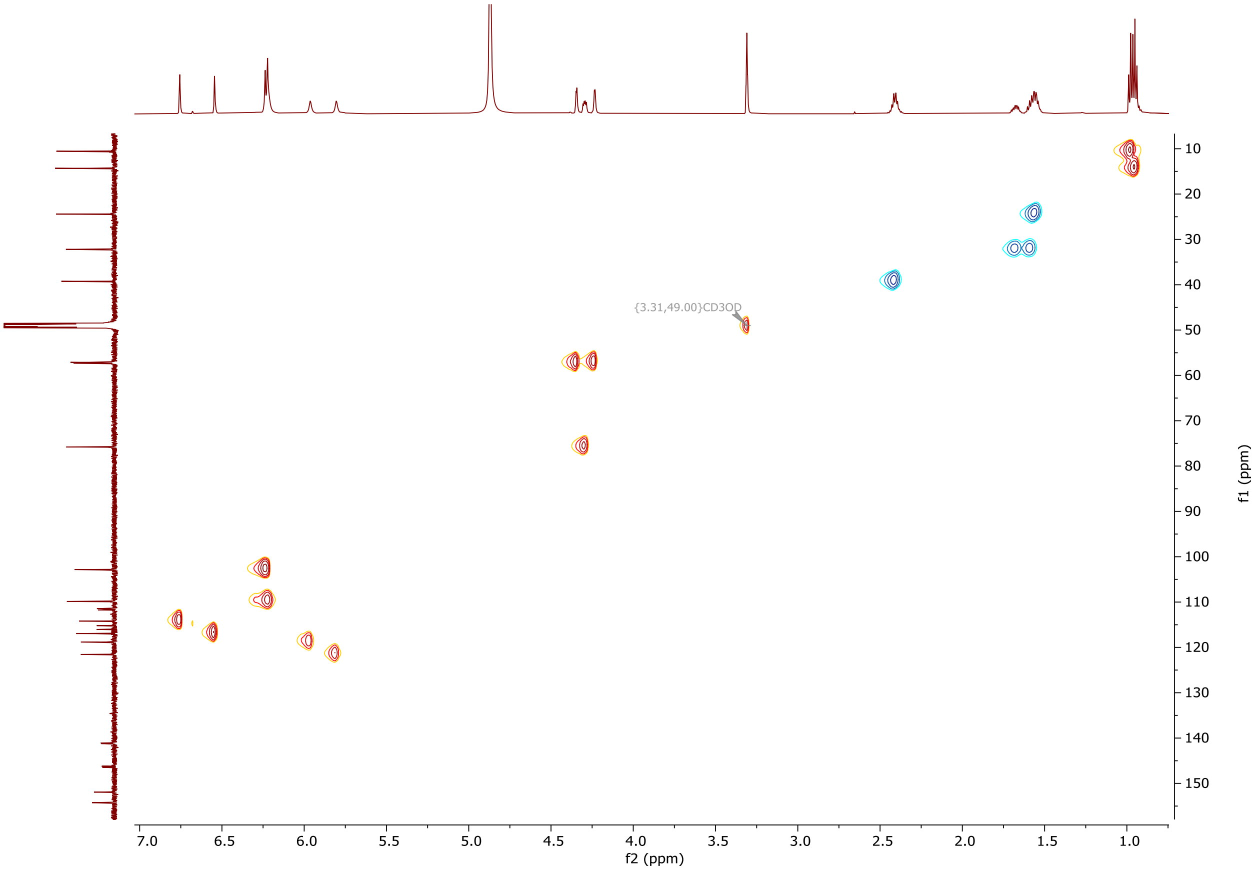

Figure S33. ^{1}H–^{13}C HSQC NMR spectrum of 1''–hydroxycrinemodin bianthrone (**3**) in MeOH-d_4. The spectrum was acquired over 64 scans at 1024 t1 increments using the standard hsqcedetgpsisp2.3 Bruker pulse sequence.

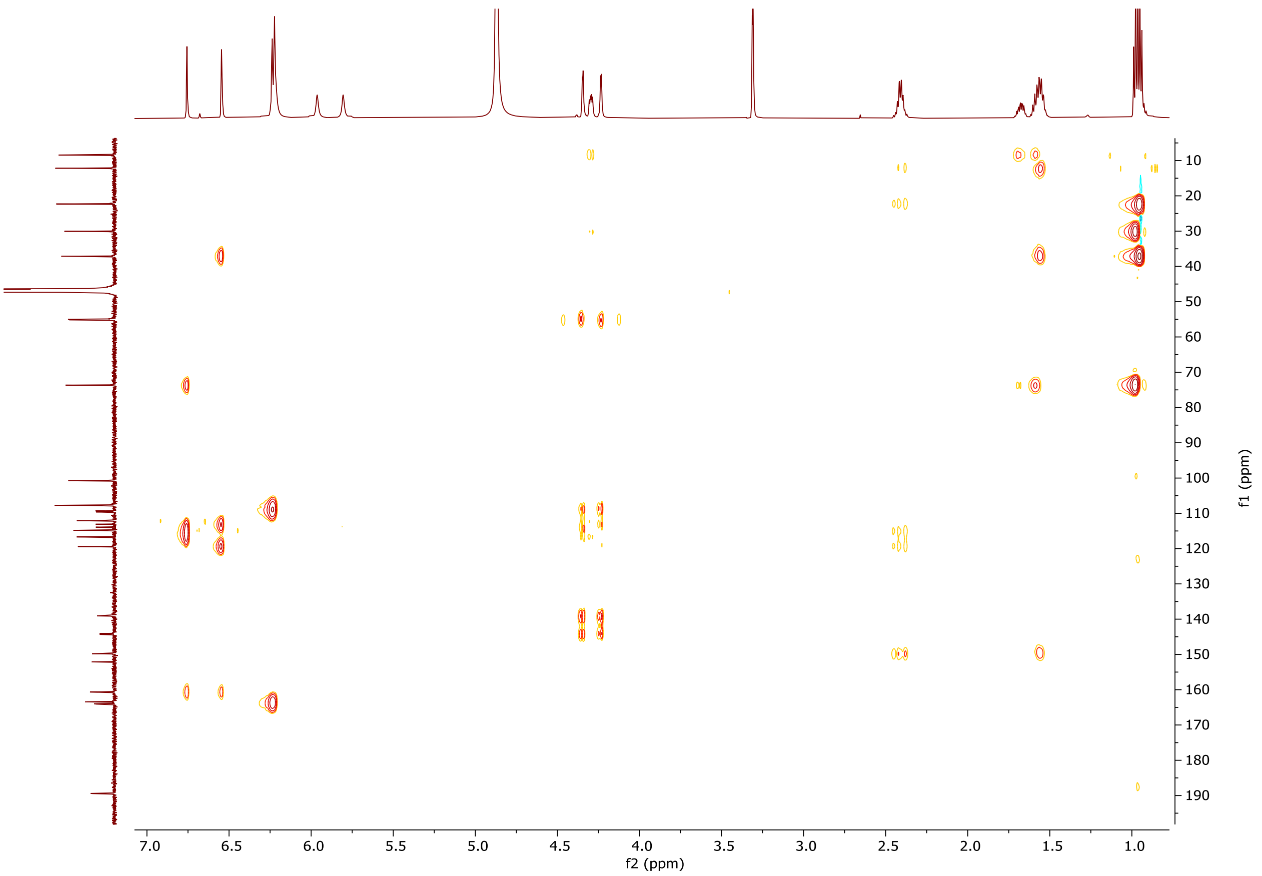

Figure S34. ^{1}H–^{13}C HMBC NMR spectrum of 1''–hydroxycrinemodin bianthrone (**3**) in MeOH-d_4. The spectrum was acquired over 240 scans at 2048 t1 increments using the standard hmbcetgpl3nd Bruker pulse sequence.

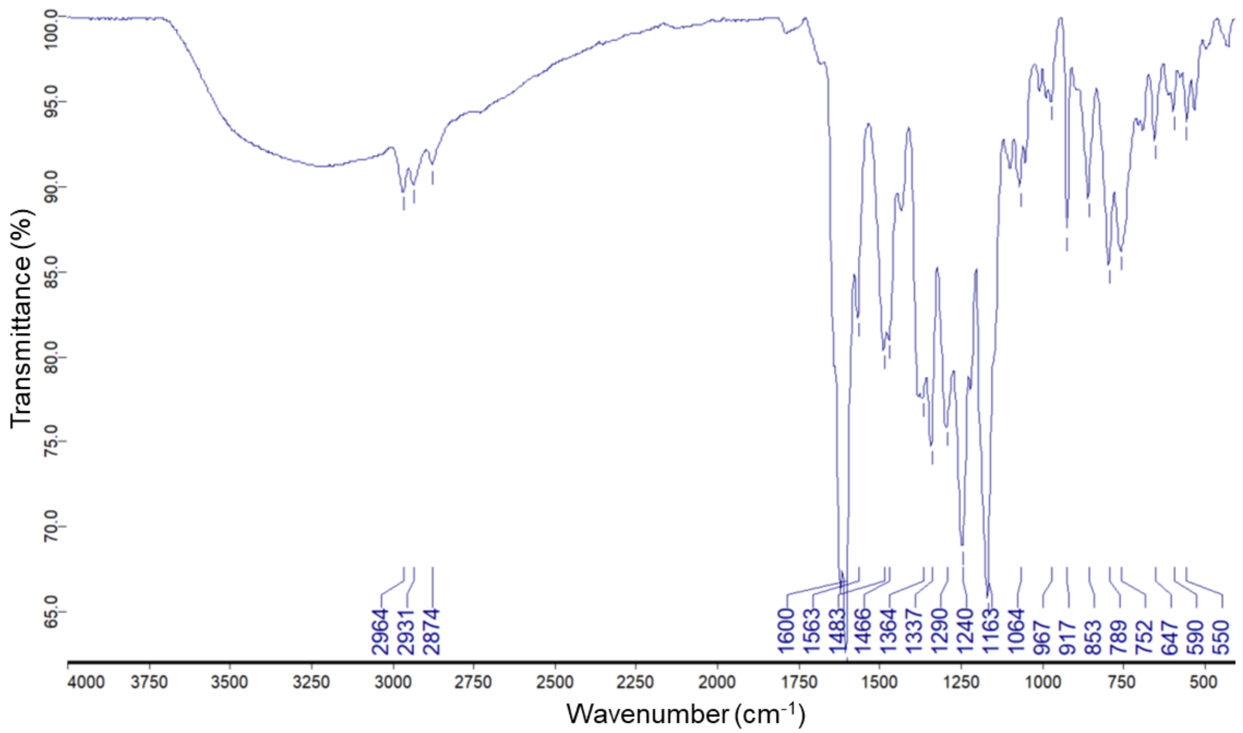

Figure S35. FTIR spectrum of 1''–hydroxycrinemodin bianthrone (**3**).

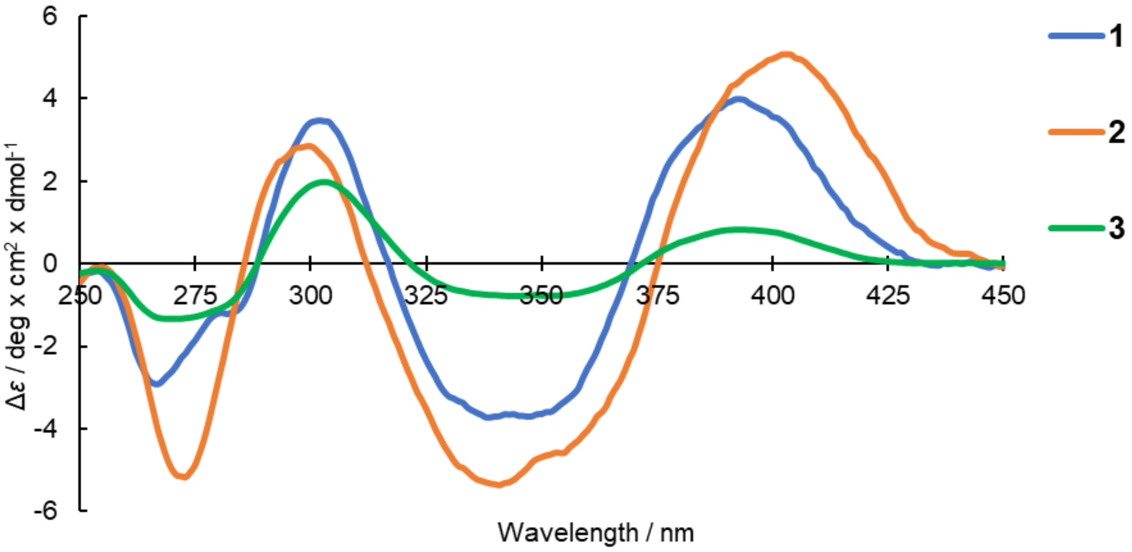

Figure S36. ECD spectra overlap of compounds **1**–**3** in acetonitrile.

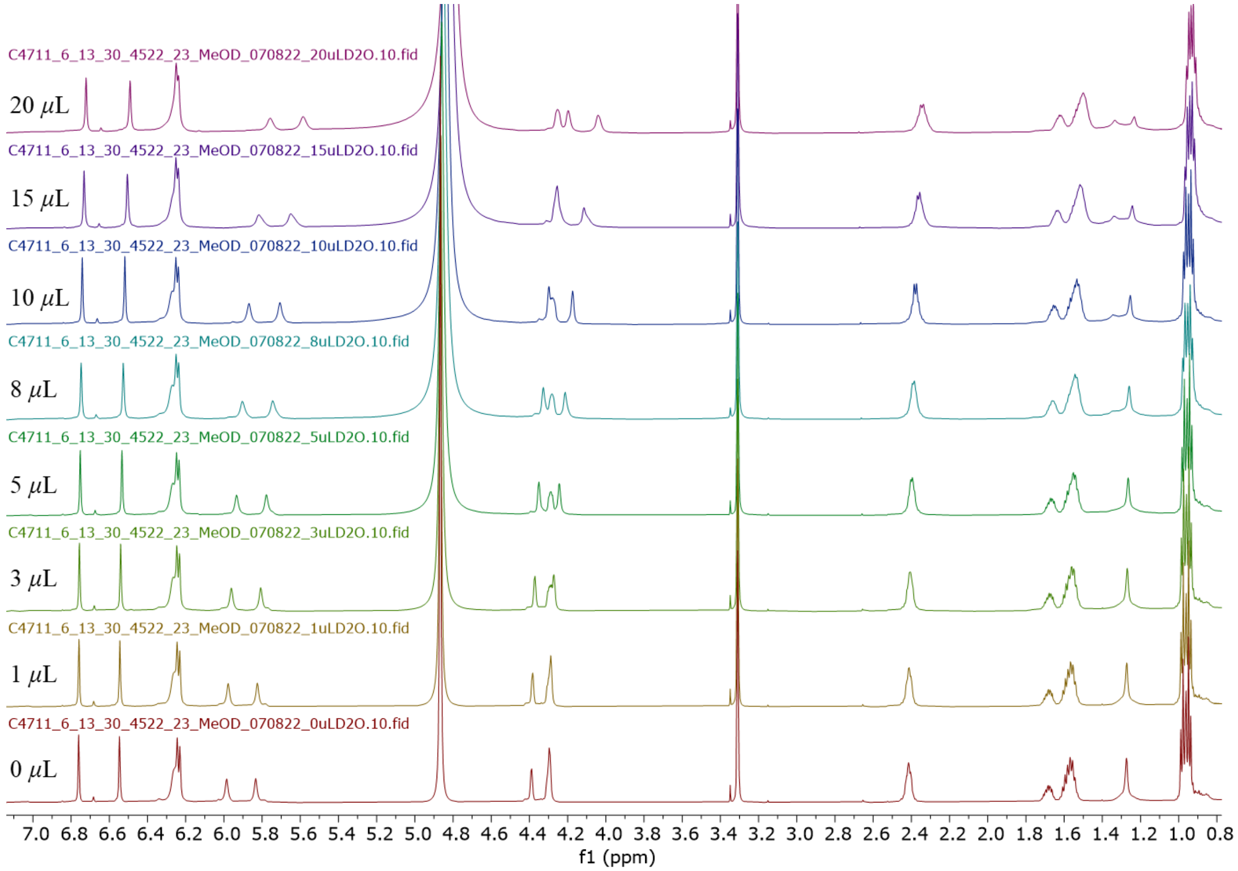

Figure S37. ^{1}H NMR spectra of **3** (in MeOH-d_4) titration with D_2O, from 0 μL (bottom) to 20 μL (top) of D_2O.

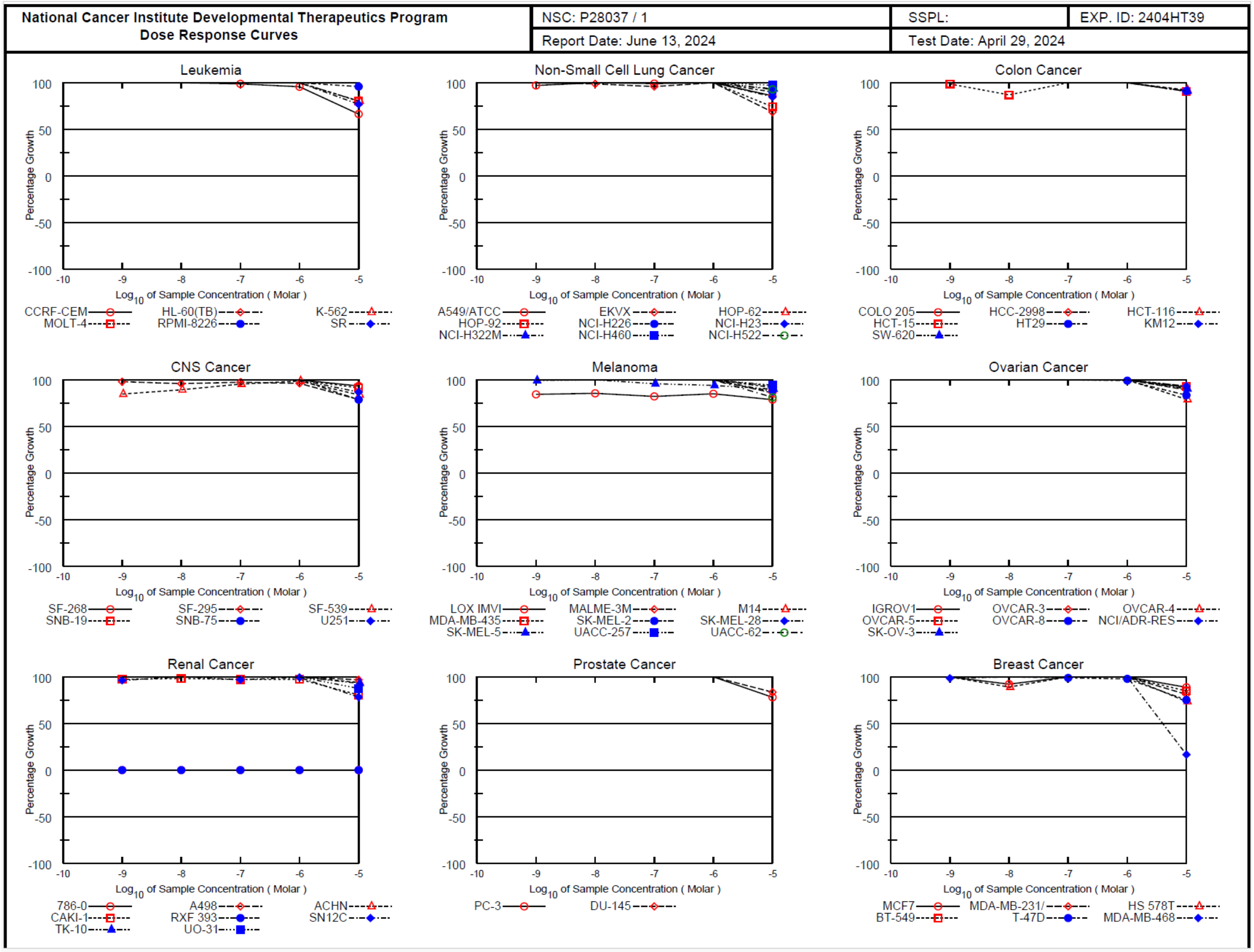

Figure S38. HTS384 NCI60 screen data for crinemodin bianthrone (**1**).

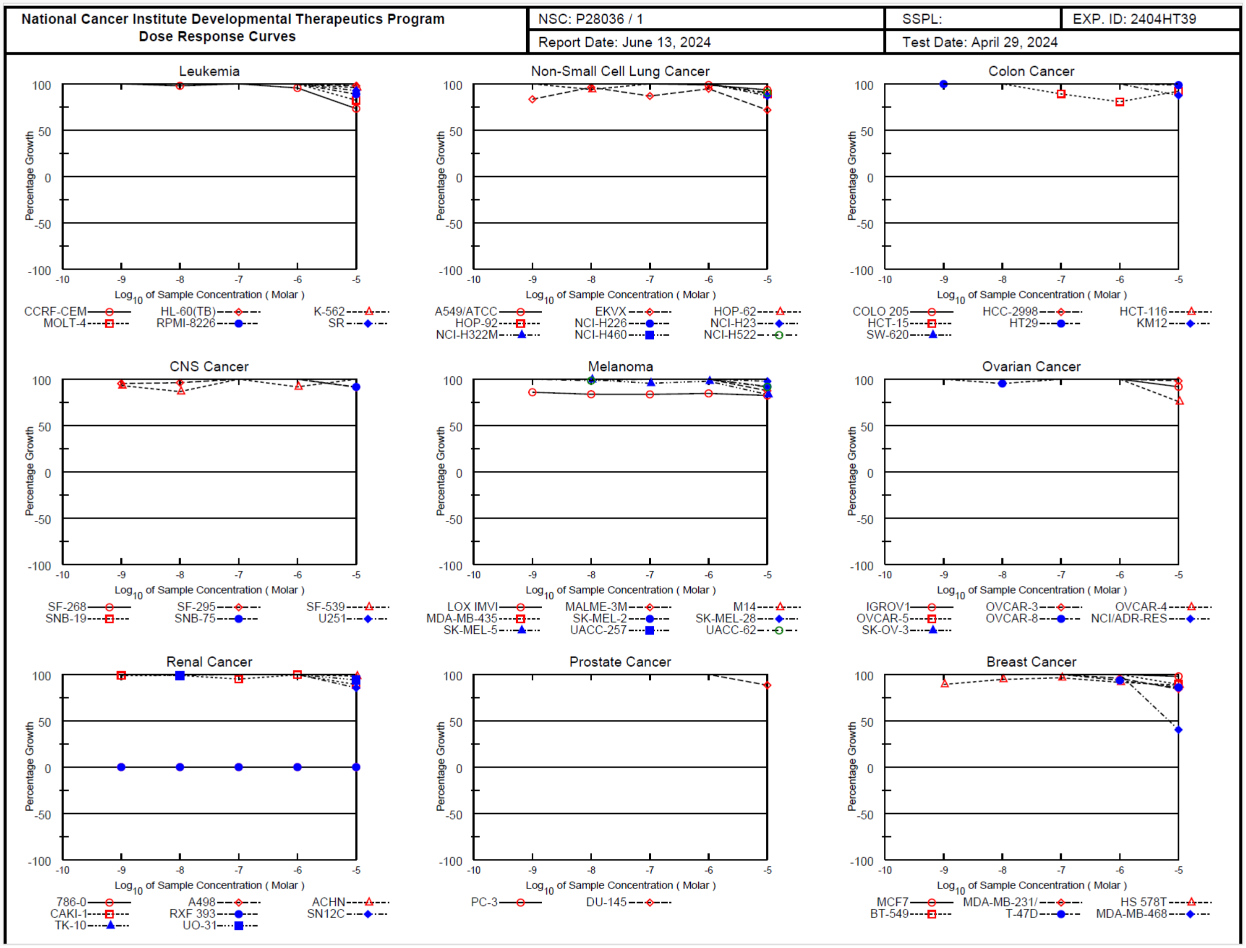

Figure S39. HTS384 NCI60 screen data for 1''–dehydrocrinemodin bianthrone (**2**).

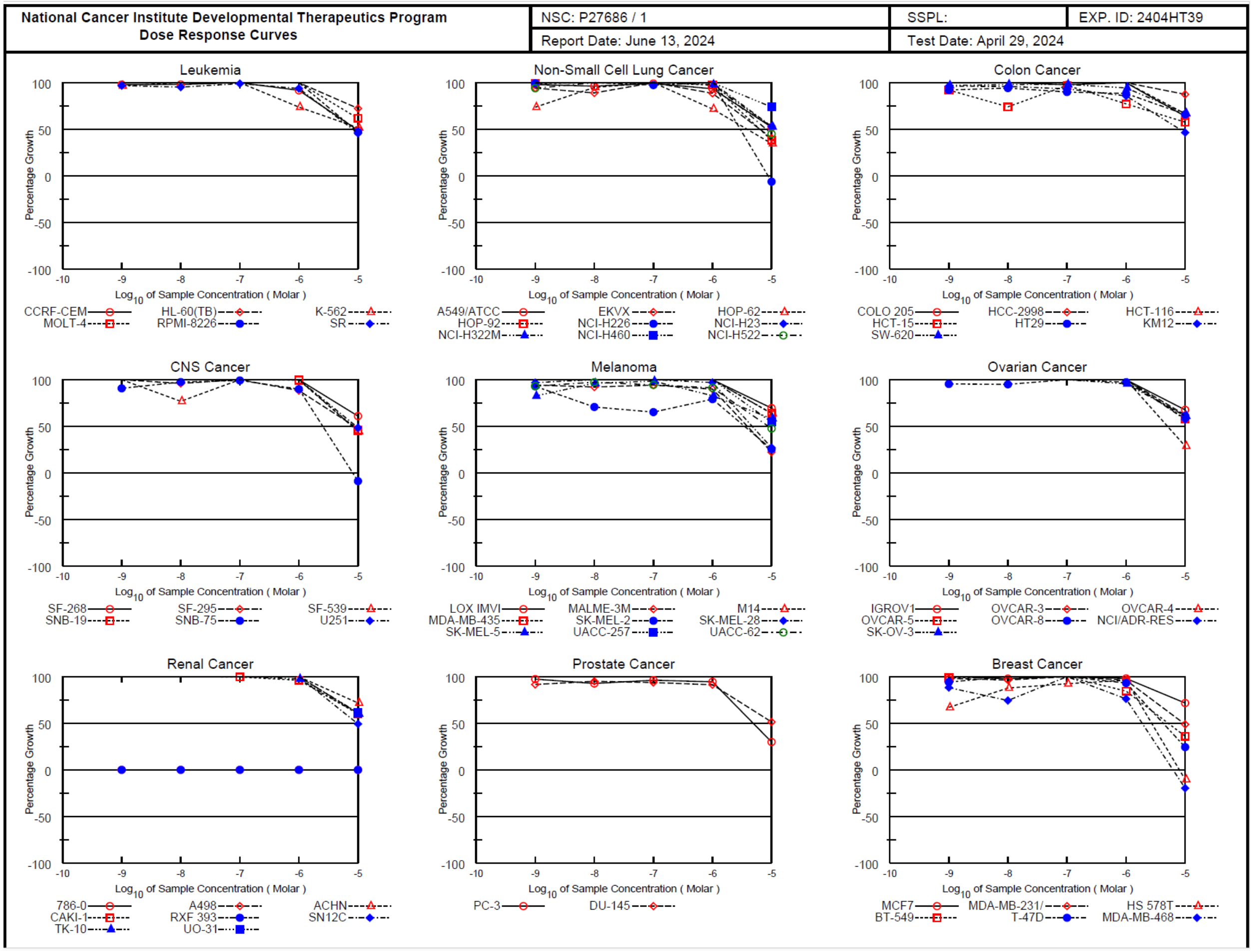

Figure S40. HTS384 NCI60 screen data for 1''–hydroxycrinemodin bianthrone (**3**).

Table S1. Reaction rates of **3** reacted with propionic anhydride using *R*- or *S*-HBTM catalysts. The reaction rate was achieved by measuring the peak areas for the fully acylated derivative, using the extracted ion chromatogram (EIC) for the sodiated molecule $[M + Na]^+$ at *m/z* 997.3617 (± 20 ppm).

**Time** (min)	***R*-HBTM** (peak area)	***S*-HBTM** (peak area)
5	5.16E+06	3.63E+06
10	1.26E+07	8.05E+06
15	1.98E+07	1.12E+07
20	2.43E+07	1.53E+07
25	3.10E+07	1.89E+07
30	3.40E+07	2.13E+07
35	3.82E+07	2.40E+07
40	4.20E+07	2.49E+07
45	4.42E+07	2.81E+07
50	4.80E+07	2.97E+07
55	5.09E+07	3.26E+07
60	5.33E+07	3.47E+07
